# Does wood mulch trigger microbially mediated positive plant-soil feedback in degraded boreal forest sites? A *post hoc* study

**DOI:** 10.3389/fpls.2023.1122445

**Published:** 2023-05-03

**Authors:** Franck Stefani, Julien Beguin, David Paré, Marie-Josée Morency, Christine Martineau, J. André Fortin, Nelson Thiffault, Armand Séguin

**Affiliations:** ^1^ Agriculture and Agri-Food Canada, Ottawa Research and Development Centre, Ottawa, ON, Canada; ^2^ Institut de recherche sur les forêts, Université du Québec en Abitibi-Témiscamingue, Rouyn-Noranda, QC, Canada; ^3^ Natural Resources Canada, Canadian Forest Service, Laurentian Forestry Centre, Québec, QC, Canada; ^4^ Université Laval, Faculté de foresterie, de géographie et de géomatique, Département des sciences du bois et de la forêt, Québec, QC, Canada; ^5^ Natural Resources Canada, Canadian Forest Service, Canadian Wood Fibre Centre, Québec, QC, Canada

**Keywords:** bacteria, belowground microbiome, fungi, plant-soil feedback (PSF), productivity, ramial chipped wood (RCW), soil nutrients, boreal forest

## Abstract

**Introduction:**

Reforestation of degraded lands in the boreal forest is challenging and depends on the direction and strength of the plant-soil feedback (PSF).

**Methods:**

Using a gradient in tree productivity (null, low and high) from a long-term, spatially replicated reforestation experiment of borrow pits in the boreal forest, we investigated the interplay between microbial communities and soil and tree nutrient stocks and concentrations in relation to a positive PSF induced by wood mulch amendment.

**Results:**

Three levels of mulch amendment underlie the observed gradient in tree productivity, and plots that had been amended with a continuous layer of mulch 17 years earlier showed a positive PSF with trees up to 6 m tall, a closed canopy, and a developing humus layer. The average taxonomic and functional composition of the bacterial and fungal communities differed markedly betweenlow- and high-productivity plots. Trees in high-productivity plots recruited a specialized soil microbiome that was more efficient at nutrient mobilization and acquisition. These plots showed increases in carbon (C), calcium (Ca), nitrogen (N), potassium (K), and phosphorus (P) stocks and as well as bacterial and fungal biomass. The soil microbiome was dominated by taxa from the fungal genus Cortinarius and the bacterial family Chitinophagaceae, and a complex microbial network with higher connectivity and more keystone species supported tree productivity in reforested plots compared to unproductive plots.

**Discussion:**

Therefore, mulching of plots resulted in a microbially mediated PSF that enhances mineral weathering and non-symbiotic N fixation, and in turn helps transform unproductive plots into productive plots to ensure rapid restoration of the forest ecosystem in a harsh boreal environment.

## 1 Introduction

Land degradation is a global threat that can lead to unproductive ecosystems under extreme conditions. Planting trees is an effective strategy for reclaiming degraded sites (Chazdon, 2008; Macdonald et al., 2015), but several abiotic and biotic factors can affect tree survival and productivity in harsh environments. Attempts to reforest degraded sites in boreal ecosystems can result in stands of stunted trees, reflecting low plant productivity, unhealthy soils, and an unsuccessful reclamation process.

The soil microbiome is fundamental to soil health and plant productivity (Saleem et al., 2019). It is essentially composed of bacterial and fungal communities that are closely associated with vascular plants (Van Der Heijden et al., 2008; Habiyaremye et al., 2020; Tedersoo et al., 2020) through plant-soil feedback (PSF) loops. In a PSF loop, plants affect the composition and activity of soil microorganisms, which in turn affect plant fitness by regulating soil nutrient fluxes (Bever et al., 1997). More specifically, belowground microbial activity and interactions contribute to the release of key plant nutrients through biochemical weathering (Smits et al., 2008; Uroz et al., 2009; Fontaine et al., 2016; Zaharescu et al., 2020), mobilization of nutrients from soil organic matter (Pritsch and Garbaye, 2011) or atmospheric nitrogen fixation (Puri et al., 2020). Within the soil microbiome, the interactions between ectomycorrhizal (EM) fungi and nitrogen-fixing bacteria are key (Li and Hung, 1987; Paul et al., 2007), as they allow the host plant to access phosphorus and nitrogen in oligotrophic environments, such as the boreal bioclimatic zone. Together, these biotic and abiotic effects influence the net outcome of PSF. The outcome is expected to be neutral or positive in ecosystems dominated by EM fungi in temperate (Bennett et al., 2017) and boreal forests (Bahram et al., 2020) or during early successional stages (Reynolds et al., 2003). Therefore, seedlings planted for the purposes of reclaiming degraded sites in boreal ecosystems should recruit these beneficial microorganisms as this will allow them to access limited nutrients supporting their growth, thus initiating a positive PSF.

However, the boreal environment is characterized by harsh micro-environmental conditions that often hinder the survival and growth of newly established seedlings. These harsh conditions are amplified in the large stripped surfaces of borrow pits, which have thermal conditions similar to frost hollows because the absence of a closed-crown canopy leads to recurrent frost events during the growing season (Plasse and Payette, 2015). In addition, newly established seedlings in borrow pits cannot benefit from the soil microbial legacy due to the absence of topsoil. The soil bank of beneficial microorganisms is only replenished by wind-dispersed epigeous fungi and bacteria (Horton, 2017; Karlsson et al., 2020) and by soil fauna (Lilleskov and Bruns, 2005; Anslan et al., 2016). As a result, plant colonization is slow and considered to be akin to primary succession, and vascular plants have difficulty becoming established (Hugron et al., 2011). Finally, the soil properties in borrow pits (oligotrophic conditions, soil compaction, low pH, low moisture level and low cation exchange capacity) limit the establishment and growth of tree seedlings (Stone and Elioff, 1998; de Souza et al., 2021). Borrow pits have poor soil structure, which can lead to strong negative feedback (Bergmann et al., 2016). Therefore, in order to successfully rehabilitate land under these challenging environmental conditions, the reforestation strategy should include treatments to induce the positive PSF expected from EM trees in boreal forests.

In 1999, a unique long-term field experiment with different forest restoration treatments was established in two borrow pits in the boreal forest on the North Shore of the Saint Lawrence river in Québec, Canada. Treatments included plots with various combinations of fertilizer applications and ramial chipped wood (RCW) amendments, while control plots were planted and left unamended. Amendments were applied either to each tree, or to each plot as a continuous layer of RCW prior to planting. Seventeen years after the start of the experiment, the control and treated plots were characterized by three levels of forest restoration success and productivity: unamended plots had null productivity, meaning no trees survived; plots amended with RCW at the tree scale had a low level of productivity, meaning some trees survived but their growth was stunted, and few reached heights greater than 1 m; and the plots amended with a continuous layer of RCW had a high level of productivity, supporting a growing forest with trees up to 6 m tall, a closed canopy, and a developing humus layer.

Many PSF experiments have been conducted under controlled conditions, and field studies have been recommended to improve the mechanistic understanding of PSF (Kulmatiski et al., 2008; van der Putten et al., 2013). Understanding how the interplay between soil nutrients and fungal and bacterial communities contributes to the conversion of unproductive plots to productive plots can help inform restoration practices in degraded boreal forests. The remarkable difference in forest reclamation and tree productivity resulting from the present field experiment allowed us to investigate the links between the taxonomic and functional diversity of the soil microbiome, soil and tree nutrient stocks and concentrations in relation to a positive PSF induced by the level of wood mulch amendment over 17 years of ecosystem development. We hypothesized that the positive PSF effects that occurred in the high productivity plots were supported by (1) contrasting microbial communities at the biomass, taxonomic and functional levels; (2) the recruitment of microbial communities specialized in mining soil for limiting soil nutrients; and (3) more interconnected fungal-bacterial networks.

## 2 Materials and methods

### 2.1 Location of borrow pits and experimental design

The field experiment was set up in two borrow pits (**Figure S1A, B**) in the North Shore region of Québec, Canada (lat. 49°51′40′′ N, long. 68°51′40′′ W) that were excavated between 1964 and 1968, leaving large stripped surfaces surrounded by boreal forest stands (**Figure S1C, D**). The harsh micro-environmental conditions, combined with the absence of topsoil, have prevented the natural recolonization of these sites over the years. The climate is humid and cold with a mean annual temperature of 1 °C and total precipitation of 950 mm. In 1998, a tractor was used to level the ground before planting. Jack pine (*Pinus banksiana*) seedlings obtained from the Centre de production de plants forestiers de Québec (provenance x05-33-92) were planted in June 1999 at a planting density of about 7,000 trees per ha. Each seedling received 25 g of Nutricote ® 18-6-8 fertilizer (Plant Products Co. Ltd., Brampton, ON, Canada, **Table S1**), with 80% of N being released within a 70-day period at 25 °C. The experimental design included two treatments and one control per block, each replicated three times, for a total of nine experimental units (hereafter plots) each covering 50 m^2^ (N = 9) and planted with 35 trees (**Figure S2A-D**). Two complete blocks were established in the first borrow pit (BP1-1 and BP1-2, 0.4 ha, **Figure S1C**), and one complete block in the second borrow pit (BP2, 0.2 ha, **Figure S1D**). The treatments (**Figure S2A-D**) were as follows: (1) plots blanketed with locally produced RCW (dry weight = 0.157 kg per L, 3 kg per m^2^, thickness = ~2-3 cm, ~150 kg ~ plot), leading to high-productivity plots (HPP); (2) plots where ~3 – 4 L of RCW was applied only at the seedling base at the time of planting with the same wood mulch thickness as in HPP but over a smaller area (~0.2 m^2^ per tree, ~19 kg per plot), resulting in low-productivity plots (LPP); and (3) unamended control plots with no surviving trees, resulting in null-productivity plots (NPP).

### 2.2 Analyses of nutrient stocks and concentrations

In September 2014, three soil stations were installed in each experimental unit to determine the nutrient stocks in the soil and tree tissues. The soil was sampled volumetrically at depths of 0–10 cm and 10–20 cm using a 4.5-cm diameter steel cylindrical corer. Samples were air-dried and sieved through a 2-mm mesh prior to analysis. On the HPP, diameter at breast height (DBH = 1.3 m) was recorded for all trees. Two trees were selected in each experimental unit to cover the full range of DBH values found on all planted plots, and they were then harvested and their stumps excavated. The branches and foliage were separated from the trunks in the field. The samples were brought to the laboratory, where they were oven-dried and weighed according to the type of material sampled (foliage, branches, trunk and stump). Second-order polynomial equations were fitted to the DBH data to estimate dry mass for each of the four material types. The DBH of all the trees was recorded to estimate the biomass and the amount of nutrients that accumulated in the trees. The nutrient content of the tree tissues was determined from samples oven-dried at 70°C. Nutrient accumulation in the soil (total N and available P and exchangeable cations) and in the vegetation during the 16 years of ecosystem development in the HPP was estimated by assuming that the NPP represented the initial conditions for all sites since time zero measurements were not available (**Table 1**). Given that these sites were very poor in nutrients and organic matter with no vegetation colonization (**Figures S2B, E**), it is reasonable to assume that the soil conditions in NPP did not evolve significantly over the 16 years of the experiment. RCW was produced from local small deciduous trees (*Alnus*, *Betula*) and its nutrient content was assessed from ten subsamples using the methods described below for vegetation. Total C content and total N content in soil and vegetation samples were analyzed using a LECO TruMac CNS analyzer (LECO Corporation, St. Joseph, MI, USA). Following calcination of plant tissues at 500°C and dilution with hydrochloric acid, cations and (P) were determined by inductively coupled plasma optical emission spectroscopy (ICP-OES) (Miller, 1997). A Mehlich III solution was used to extract base cations and available P (Carter and Gregorich, 2007), which were determined by ICP-OES. Finally, soil samples were also collected simultaneously with samples used to characterize the soil microbiome (see below) during the sampling campaign conducted in October 2016. Soil nutrients were measured as previously described, and pH was analyzed in distilled water and in 0.01M CaCl_2_.

**Table 1:** Estimated nutrient stocks in added RCW (time zero), soil and tree tissues (year 16) in null-productivity plots (NPP) and high-productivity plots (HPP). Units are in g m^-2^ unless otherwise noted.

	Site	Productivity	Nitrogen	Phosphorus	Potassium	Calcium		Magnesium	
RCW		HPP	4.82	0.67	3.61	13.38		1.10	
Soil	BP1-1	HPP	2.94	4.49	0.44	1.25		0.19	
	BP1-2	HPP	2.72	4.67	0.54	2.05		0.35	
	BP2	HPP	2.07	4.12	0.65	1.54		0.35	
	BP1-1	NPP	2.19	2.84	0.25	0.47		0.16	
	BP1-2	NPP	1.94	3.47	0.23	0.49		0.17	
	BP2	NPP	1.96	1.63	0.41	1.76		0.54	
Tree	BP1-1	HPP	16.81	2.56	7.19	12.25		1.68	
	BP1-2	HPP	17.97	2.78	7.83	13.35		1.83	
	BP2	HPP	23.77	3.56	9.97	16.98		2.32	
Soil + tree	BP1-1	HPP	19.75	7.05	7.63	13.50		1.87	
	BP1-2	HPP	20.69	7.45	8.36	15.40		2.18	
	BP2	HPP	25.84	7.67	10.62	18.51		2.66	
Difference between HPP and NPP ([soil + tree] - soil)			17.56	4.21	7.38	13.03		1.71	
			18.75	3.98	8.13	14.91		2.02	
			23.89	6.05	10.21	16.75		2.12	
Average nutrient accumulation over 16 years (g m^-2^)			20.07	4.75	8.57	14.90		1.95	
Average nutrient accumulation per year (kg ha^-1^ y^-1^)			12.54	2.97	5.36	9.31		1.22	

### 2.3 DNA isolation and amplification

To characterize the soil microbiome, bulk soil, rhizosphere and roots were sampled at the end of October 2016. In the LPP and HPP, soil cores were collected at each cardinal point around three randomly selected trees (sampling unit), at a distance of 30 cm from the base of the trunks. In the NPP, soil cores were collected at each cardinal point around three randomly selected points because no trees had survived (**Figures S2E-G**). The roots in the soil cores from the HPP and LPP were separated from the bulk soil. Sieved bulk soil and root-attached soil (i.e., rhizosphere) were used to characterize the soil and rhizosphere compartments, respectively (**Figure S3**). Samples were kept in a cooler during fieldwork and were stored at –80 °C in the laboratory until DNA extraction.

In the step prior to DNA extraction, root fragments were cleaned in 150-ml glass test tubes containing 15 ml of sterile phosphate buffered saline (PBS) solution (1X). Samples were vortexed for 10–15 s and sonicated 5 times for 30 s with a 30 s interval between each sonication. This step was repeated using new tubes and fresh PBS 1X solution. Cleaned root fragments were removed from the tubes, blotted dry and cut up. Up to 100 mg of roots were then weighed and placed in a 2-ml screw cap tube with a 5-mm stainless steel bead. The roots were flash frozen in liquid nitrogen and ground into a fine powder using a TissueLyzer II (QIAGEN, Valencia, CA, USA) at 26 Hz for 2 × 45 s. DNA was then extracted from the powder using the MO BIO PowerSoil DNA Isolation Kit (Mo Bio Laboratories Inc., Solana Beach, CA, USA). A total of 250 mg of starting material was used to isolate DNA from bulk soil and rhizosphere soil using the same extraction kit. Crude genomic DNA (gDNA) extracts were quantified using a Qubit Fluorometer 2.0 (Life Technologies, Burlington, ON, Canada) and the Qubit dsDNA HS assay kit. The concentration of gDNA was standardized by diluting each sample to 5 ng/μl (samples with concentrations lower than 5 ng/μl were not diluted).

Primer pair 515F-Y (5’-GTGYCAGCMGCCGCGGTAA-3’) and 926R (5’-CCGYCAATTYMTTTRAGTTT-3’) (Parada et al., 2016) was used to amplify the bacterial V4–V5 regions of the nuclear 16S ribosomal gene. The primer pair ITS9 (5’-GAACGCAGCRAAIIGYGA-3’) (Menkis et al., 2012) and ITS4 (5’-TCCTCCGCTTATTGATATGC-3’) (White et al., 1990) was used to amplify the fungal ITS2 region of the nuclear ribosomal gene. The amplification was performed in 25 µl of reaction mix in triplicate as follows: 9 μl of UltraPure™ Dnase/Rnase-Free distilled water (GIBCO, life technologies), 200 µM of each dNTP, 1.5 mM of Mg^2+^, 200 NT of each primer, 1 U of HotStarTaq *Plus* DNA Polymerase (Qiagen, Valencia, CA, USA) and ~ 12.5 ng of gDNA. Thermocycling conditions were as follows: initial denaturation step at 95°C for 5 min, 34 cycles at 94°C for 30 s, 50°C for 30 s, and 72°C for 1 min, and a final elongation step at 72°C for 10 min.

### 2.4 High-throughput sequencing

For high-throughput multiplexing and sequencing of amplicons, triplicate PCR products from each sample were pooled, purified and indexed. Residual dNTPs, primers and buffers were removed in a purification step using 81 μl of Agencourt AMPure XP beads (Beckman Coulter Inc., Indianapolis, IN, USA) according to the protocol described in Illumina’s *16S Metagenomic Sequencing Library Preparation Guide* (Part # 15044223 Rev. B). Purified amplicons were indexed in 50 µl of the following reaction mixture: 10 μl of UltraPure™ Dnase/Rnase-Free distilled water, 5 μl of each Nextera XT v2 Index Primer (Illumina Inc., San Diego, CA, USA), 25 μl of KAPA HiFi HotStart Ready Mix (KAPA Biosystems, Wilmington, MA, USA), and 5 μl of the purified PCR product. Thermocycling conditions were as follows: initial denaturation step at 95°C for 3 min, 8 cycles of 95°C for 30 s, 55°C for 30 s, 72°C for 30 s, and a final elongation step at 72°C for 5 min. Indexed amplicons were purified using 56 μl of Agencourt Ampure XP beads as described above, quantified using a Qubit Fluorometer 2.0 with the Qubit dsDNA BR assay kit, and multiplexed using an equimolar ratio. Paired-end sequencing (2 × 250 bp) of the 16S/ITS pools was performed on an Illumina MiSeq System sequencer and the MiSeq Reagent Kit v2 (500 cycles) on the Nucleic Acid Solutions Group’s Illumina sequencing platform at the Aquatic and Crop Resource Development Research Centre, National Research Council Canada, Saskatoon, SK, Canada. The Illumina data generated in this study have been deposited in the National Center for Biotechnology Information’s (NCBI’s) Sequence Read Archive under accession number PRJNA775865.

### 2.5 Analyses of the microbial biomass and *nifH* abundance

Bacterial and fungal biomass in the rhizosphere and bulk soil and the abundance of the *nifH* gene, which encodes the nitrogenase enzyme and is responsible for N_2_ fixation in bacteria, were measured using a qPCR assay. Primer pair 27F (5’-AGAGTTTGATCMTGGCTCAG-3’) and 518R (5’-TTACCGCGGCTGCTGG-3’) (Okubo et al., 2015) was used to amplify the 16S rRNA gene. The primer pair FungiQuant-F (5’-GGRAAACTCACCAGGTCCAG-3’) and FungiQuant-R (5’-GSWCTATCCCCAKCACGA-3’) (Liu et al., 2012) was used to amplify the 18S rRNA gene. The primer pair Ueda19F (5’-GCIWTYTAYGGIAARGGIGG-3’) and UedaR6 (5’-AAICCRCCRCAIACIACRTC-3’) (Angel et al., 2018) was used to amplify the *nifH* gene. The 10 μl reaction mix consisted of 5 μl of 2× QuantiTect SYBR Green PCR Master Mix, 2 μl of a 5 μM primer mix, 1 μl of Rnase-free water, and 1 μl of DNA extract (~ 0.5–5 ng of DNA). Thermocycling conditions were as follows: initial denaturation step at 95°C for 15 min; 40 cycles at 95°C for 15 s; 30 s at 54°C for the 16S rRNA gene, at 59°C for the 18S rRNA gene and at 52°C for the *nifH* gene; and 65°C for 90 s. Fluorescence measurements were performed at the end of the elongation step of each cycle, and the specificity of each reaction was verified by melting curve analysis. Standard curves were used to determine the copy number of each gene in the DNA extracted from the rhizosphere and bulk soil. The curves were calculated using a 10-fold dilution series of quantified DNA from pure cultures of *Sinorhizobium meliloti* 1607 (for the 16S rRNA and *nifH* genes) and *Ophiostoma novo-ulmi* H327 (for the 18S rRNA gene). For all assays, qPCR was performed on an ABI 7500 apparatus (Applied Biosystems; Thermo Fisher Scientific Inc., Waltham, MA, USA) using a QuantiTect SYBR Green PCR Kit (QUIAGEN, Valencia, CA, USA) according to the manufacturer’s instructions.

### 2.6 Bioinformatic analyses

To generate microbial species tables using the raw demultiplexed sequences, paired-end sequences were first trimmed, truncated, assembled, filtered for chimeras, and denoised using the DADA2 plugin (Callahan et al., 2016) as established in QIIME 2 v2019.1.0 (Bolyen et al., 2019). Sequences with median quality scores below 37 were trimmed. The quality scores of the ITS2 sequences from soil samples decreased from positions 140–160 bp onwards, preventing the assembly of the forward and reverse sequences. Therefore, only the forward sequences were included in the analysis of the soil fungal community. For the libraries from root and rhizosphere samples, the 3’ends of the forward and reverse sequences were truncated, and the average length of the assembled sequences was 410–415 bp, with a 21–26 bp and 50 bp overlap in the 16S and ITS2 sequences, respectively.

Amplicon sequence variants (ASVs) were generated by clustering 16S sequences (pairwise similarity threshold of 100%), and operational taxonomic units (OTUs) were generated by clustering ITS sequences (pairwise similarity threshold of 97%) as proxies for bacterial and fungal species, respectively. The number of sequences used to train the error model was set to 2,000,000. ASVs or OTUs with a frequency of less than 0.1% of the mean sample depth were filtered out, which excluded the results caused by the MiSeq bleed-through between runs as reported by Illumina (https://github.com/LangilleLab/microbiome_helper/wiki/Amplicon-SOP-v2-(qiime2-2019.7)#41-filter-out-rare-asvs). *De novo* clustering was performed using VSEARCH (Rognes et al., 2016) in QIIME 2 to cluster the fungal ITS2 sequences (97% similarity threshold) into OTUs. Taxonomic information was assigned using a out Bayes classifier (Wang et al., 2007) that was pre-trained using the Greengenes (version 13_8, files clustered using a 99% similarity threshold, McDonald et al., 2012) and UNITE (version 8.0, released on 04/02/2020, Nilsson et al., 2019) databases for bacterial and fungal datasets, respectively. Each dataset was then filtered based on taxonomic criteria. A total of 184 ASVs that were identified as ‘Archaea’, ‘Chloroplast’ and ‘Mitochondria’ were discarded from the 16S dataset. A total of 115 unassigned and non-fungal OTUs were filtered out from the ITS2 dataset. **Figure S4** shows how the number of samples, sequences, bacterial ASVs, and fungal OTUs changed during the bioinformatics processes, from raw data to ASV/OTU tables used for downstream community ecology analyses.

### 2.7 Community composition, alpha- and beta-diversity analyses

Rarefied ASV/OTU tables (**Figures S5, S6**) were used to calculate diversity indices and relative abundances of bacteria and fungi. Alpha diversity (within-sample diversity) was calculated using the sample-size- and coverage-based approaches for the rarefaction and extrapolation of the ASV/OTU-generated Hill numbers (i.e., richness, q = 0; Shannon diversity, q = 1, the exponential of Shannon entropy; and Simpson diversity, q = 2, the inverse of Simpson concentration) in the R package for iNEXT v2.0.19 (Chao et al., 2014; Hsieh et al., 2016). The Hill numbers, or effective species numbers, are related to Renyi’s definition of a generalized entropy formula, and they differ only by an exponent *q*, which determines the sensitivity to rare (low *q* values) and abundant species (high *q* values) (Hill, 1973). Faith’s phylogenetic diversity (Faith and Baker, 2006) was calculated using a subset of the fungal dataset that included only OTUs identified as agaricomycetes. For the bacterial and agaricomycetes datasets, the QIIME 2 pipeline align-to-tree-mafft-fasttree in the phylogeny plugin was run to align the sequences using MAFFT (Katoh et al., 2002) and to produce a phylogenetic tree (root: midpoint) using FastTree (Price et al., 2010). The phylogenetic trees were then used as input to calculate Faith’s phylogenetic diversity. To generate the bacterial and fungal taxonomic profiles for the NPP, LPP, and HPP, the relative abundances of ASVs and OTUs were calculated, and ASVs and OTUs with a relative abundance of less than 1% were removed. Bacterial and fungal functions were predicted using the Functional Annotation of Prokaryotic Taxa (FAPROTAX) database (www.zoology.ubc.ca/louca/FAPROTAX) (Louca et al., 2016) and FUNGuild (http://www.funguild.org) (Nguyen et al., 2016), respectively. Only guild assignments that were ‘highly probable’ and ‘probable’ were considered to avoid overinterpretation of the ecological data (Nguyen et al., 2016).

### 2.8 Analyses of differentially abundant taxa between high- and low-productivity plots

To identify differentially abundant bacteria and fungi in LPP and HPP, a differential ranking procedure was applied to the non rarefied ASV/OTU matrices using Songbird v1.0.3 (Morton et al., 2019) as established in QIIME 2. Because Songbird is sensitive to sparsity, only ASVs and OTUs that were observed with a total abundance of more than 5,000 sequences across all samples and that were found in at least 10 samples from LPP and HPP were considered. Songbird allows for the identification of differentially abundant features while accounting for the compositional nature of microbiome data and accounting for confounding variables in the datasets. Differentials are defined as the estimated log-fold change in the abundance of features across different sample types. Multinomial regressions were performed while incrementing the number of covariates of interest (treatment, soil compartment, site) in the model. The convergence statistics (graph of the prediction accuracy of the model and graph of the loss function) generated by running multinomial regressions were visualized and analyzed using QIIME 2 Visualization. Parameters (covariates, number of iterations, number of samples assessed per training iteration, width of normal prior for the differentials) were adjusted to obtain a reasonable fit. The retained model was then validated against the null and baseline models. Qurro v0.7.1 (Fedarko et al., 2020), as implemented in QIIME 2, was used to visualize rankings and sampled plots and to explore feature rankings and log-ratios.

### 2.9 Core microbiome and network analyses

The bulk soil, rhizosphere and root datasets were combined prior to performing the core microbiome analyses. Core bacterial and fungal communities (**Figure S8**) were calculated using the rarefied datasets (raw abundance) and the *core* and *plot_core* functions in microbiome R package v1.8.0 (Lahti and Shetty, 2019). Detection and prevalence thresholds were set to 0 and 70, respectively. Interactions between bacterial ASVs and fungal OTUs in LPP and HPP were modeled as Poisson log-normal (PLN) networks (Chiquet et al., 2019a; Chiquet et al., 2021) using the PLNnetwork function in R package *PLNmodels v0.10.6*. Compared to other models, the Poisson Log-Normal models have the advantage of accounting for confounding covariates and differences in sampling effort, while correcting for the compositional nature of count data, and integrating different data sets into the same network (i.e., bacterial and fungal communities in this study). Model selection was performed by incrementally adding covariates, and the model with the lowest Bayesian Information Criterion (BIC) was selected for the PLN inference. The relative log expression (RLE) normalization scheme was applied to compute scaling factors used as offsets during PLN inference and to account for differences in sequencing depth between samples.

### 2.10 Statistical analyses and data visualization

To compare the means of Hills numbers (q = 0, q = 1, q = 2) and Faith’s phylogenetic diversity from each level of productivity, Poisson (q = 0) and linear (q = 1, q = 2, and Faith’s phylogenetic diversity) mixed ANOVA models were fitted separately for each compartment (soil, rhizosphere, root). ‘Block’ and ‘restoration treatments nested within block’ were defined as nested random intercepts, and ‘restoration and ecosystem productivity level’ as fixed effects, using the lme4 package (Bates et al., 2015).

Statistical differences in the average composition of bacterial and fungal communities between (1) productivity levels (NPP, LPP, HPP), (2) the compartments (soil, rhizosphere, root), and (3) their interactions were tested using permutational multivariate analysis of variance (PerMANOVA) based on Bray-Curtis dissimilarities calculated with log(x+1)-transformed abundance data (Anderson et al., 2008). Note that because only mineral soil was available in the NPP, this interaction term was calculated only for the LPP and HPP. To test for significant differences in multivariate dispersion between groups (i.e., beta diversity), permutational analyses of multivariate dispersion (PERMDISP) were used with log(x+1)-transformed abundance data and with the same factors used above for PerMANOVA analyses (Anderson et al., 2008). Finally, we used distance-based redundancy analyses (dbRDA) to analyze the relationship between multivariate bacterial and fungal abundance data (similarity matrix) and continuous soil chemical variables measured in the 20 cm topsoil layer. PerMANOVA, PERMDISP and dbRDA analyses were all performed using the Primer 7 software (Anderson et al., 2008).

To test whether concentrations of each soil nutrient differed among productivity levels, we first fitted linear mixed models with ‘block’ and ‘restoration treatments nested within blocks’ as nested random intercepts, and ‘productivity level’ as a fixed effect. Second, when the treatment effect was significant (or close to being significant), we performed Tukey’s multiple comparison test. Statistical analyses were performed in R v3.6.3 (R Core Team, 2020) using the packages *nlme v3.1-152* (Pinheiro et al., 2020), *multcomp* (Hothorn et al., 2008), *lsmeans v2.30* (Lenth, 2016), and *lme4 1.1-27* (Bates et al., 2015).

A Poisson generalized linear mixed-effect model was used to assess the effect of forest restoration success and productivity on each gene copy number separately for each compartment, with ‘block’ and ‘restoration treatments nested within blocks’ as nested random intercepts and ‘productivity levels’ as a fixed effect. Pairwise comparisons of means were made using Tukey’s test. Statistical analyses were performed in the R package using *lme4*.

Data visualization (bar plots, non-metric multidimensional scaling [NMDS] biplots, core microbiomes, and networks) was performed using R and the following packages: *corrplot v0.84* (Wei and Simko, 2017), *ggplot2 v3.3.2* (Wickham, 2016), *ggthemes v4.2.0* (Arnold, 2021), *graphics v3.6.3* (R Core Team, 2020), *gridExtra v2.3* (Auguie, 2017), *igraph v1.2.5* (Csardi and Nepusz, 2006), *knitr v1.29* (Xie, 2014; Xie, 2015; Xie, 2022), *microbiome v1.8.0* (Lahti and Shetty, 2019), *phyloseq v1.30.0* (McMurdie and Holmes, 2013), *PLNmodels v0.10.6* (Chiquet et al., 2018; Chiquet et al., 2019a; Chiquet et al., 2019b; Chiquet et al., 2021), *plyr v1.8.6* (Wickham, 2011), *reshape2 v1.4.4* (Wickham, 2007), *rmarkdown v2.3* (Xie et al., 2018; Xie et al., 2020; Allaire et al., 2022), and *tidyr v1.1.0* (Wickham and Girlich, 2022).

## 3 Results

### 3.1 Structure and biomass of the underground microbiome

**Figure 1:**
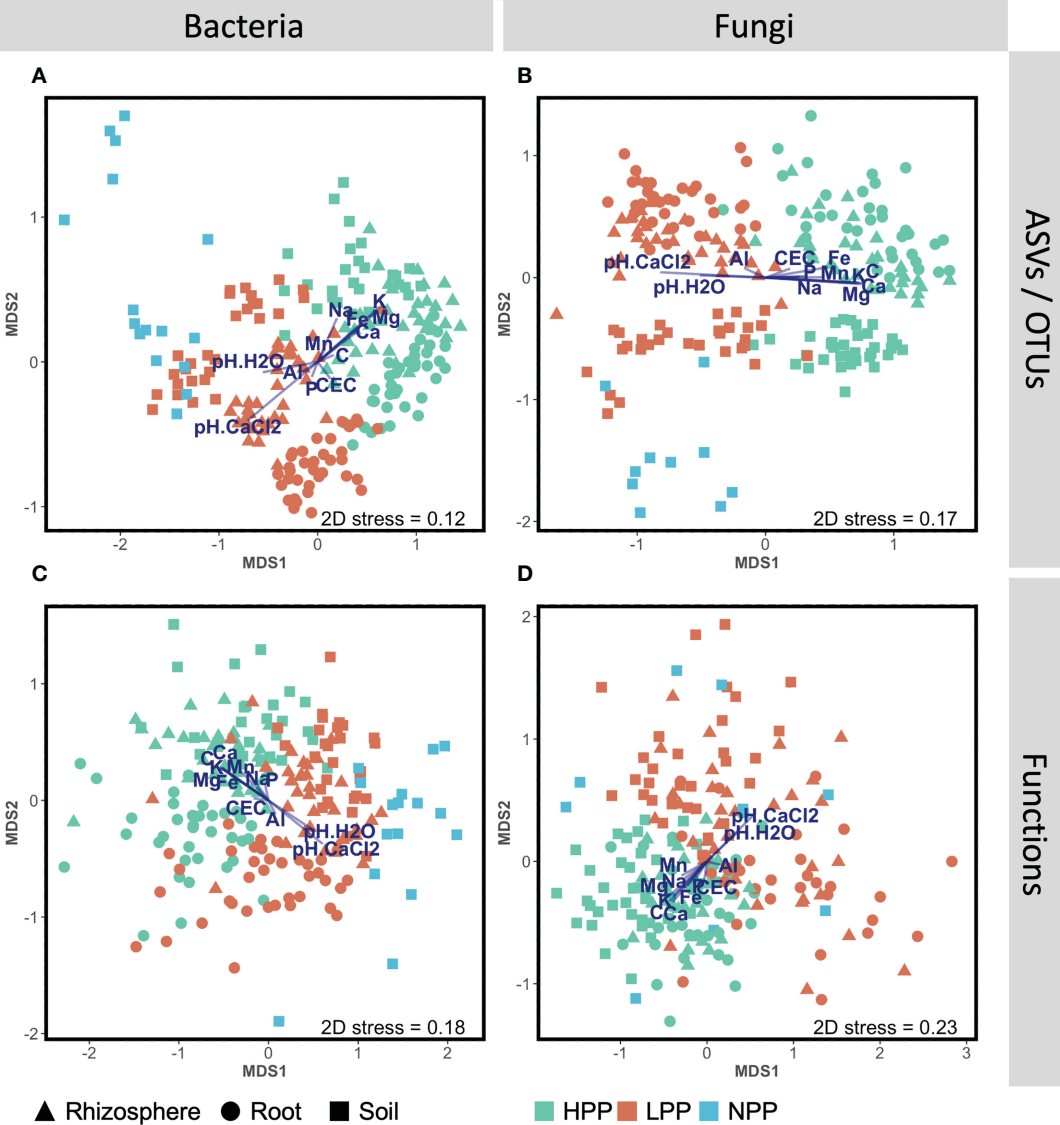
Non-metric multidimensional scaling (NMDS) ordinations showing changes of community composition between productivity levels (HPP, high-productivity plots; LPP, low-productivity plots; NP, null-productivity plots) and compartments (bulk soil, rhizosphere and roots), at the taxonomic **(A, C)** and functional **(C, D)** levels for the bacterial and fungal assemblages, based on Bray-Curtis dissimilarities calculated with log(x+1) transformed abundance data. The segment overlay represents the element concentrations in the topsoil (0–20 cm) that correlate significantly (*p*< 0.05) with the bacterial and fungal abundance data based on dbRDA analyses (see section 2.10).

**Figure 2:**
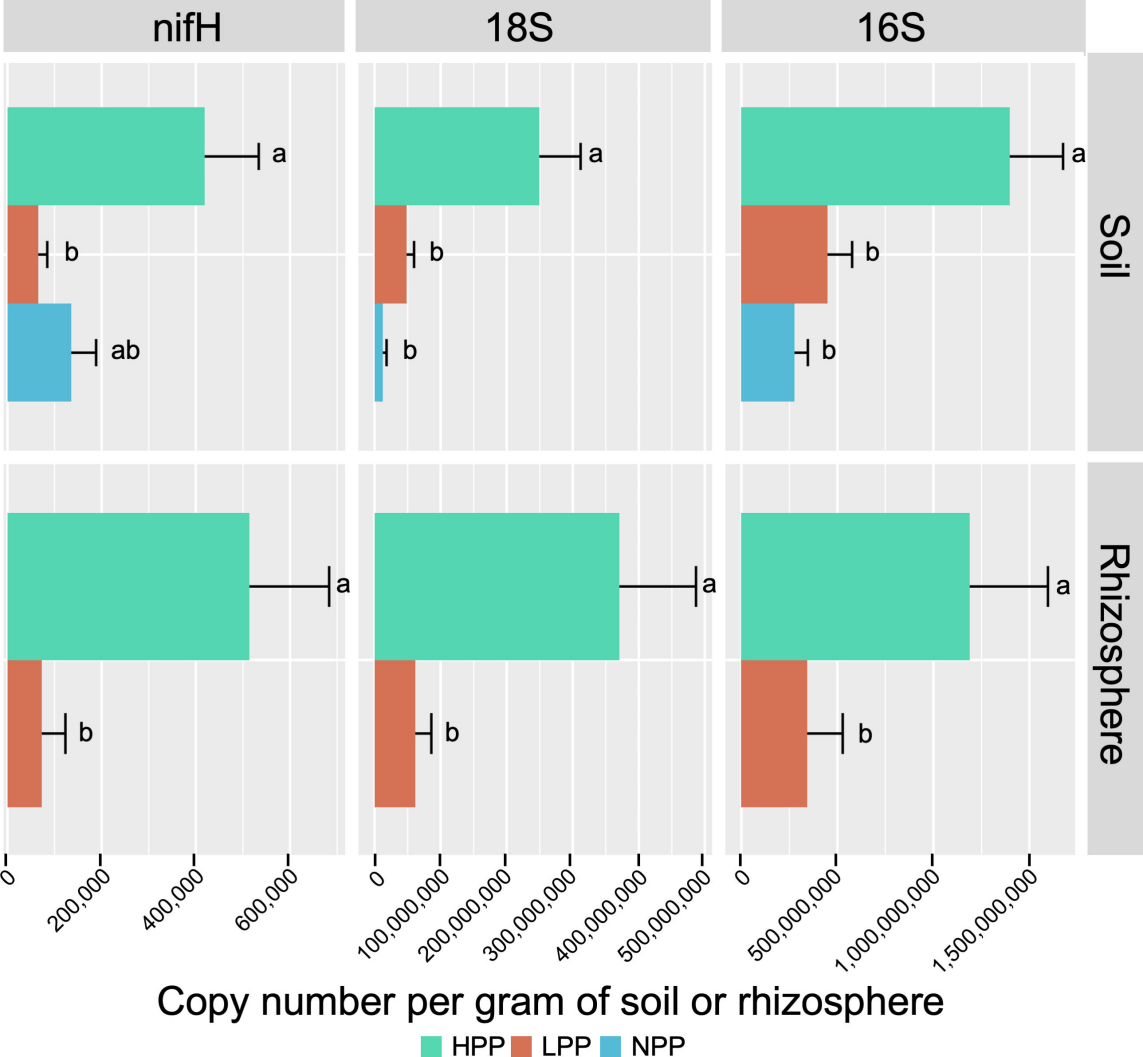
Copy number of *nifH*, 18S and 16S genes per gram of soil or rhizosphere sample, quantified by real-time PCR. Error bars are standard error of the mean. HPP, high-productivity plots; LPP, low-productivity plots; NPP, null-productivity plots.

Each of the three levels of forest restoration success and productivity produced a unique belowground microbiome (**Figure 1**) with contrasting levels of microbial biomass (**Figure 2**). The average taxonomic and functional composition of the bacterial and fungal communities differed markedly between the LPP and HPP (**Table 2**), and this effect depended on the compartments (*p*< 0.01 for most interaction terms ‘Treatment × compartment’). The level of plot productivity (LPP vs. HPP) had a significant effect on the average taxonomic and functional composition within each compartment at *p<* 0.005 for most pairwise comparisons. The largest differences were observed in the rhizosphere for bacteria (SS = 40659; *p*< 0.001) and fungi (SS = 36472; *p*< 0.001), and in the bulk soil for fungal functions (SS = 12209; *p* = 0.006, **Table 2**). No significant or biologically relevant interaction was detected for the variance in the taxonomic and functional composition (i.e., beta diversity) of the bacterial and fungal communities (**Table S2**). Therefore, the observed differences in community composition between plots are due to differences in average composition rather than differences in variance.

**Table 2:** Multivariate analysis of variance by permutation (PERMANOVA) based on Bray-Curtis dissimilarities using abundance data (log(x+1) transformed) for bacteria (ASVs and functions) and fungi (OTUs and functions) in relation to the productivity levels (null-, low-, and high-productivity plots) and compartments sampled (soil, rhizosphere, and roots).

	Bacteria ASVs				Fungi OTUs				Bacteria functions				Fungi functions			
Sources of variation	df	SS	*F*	*p*	df	SS	*F*	*p*	df	SS	*F*	*p*	df	SS	*F*	*p*
Prod	2	109780	3.9	**0.003**	2	112550	4.9	**0.003**	2	7768	6.2	**0.007**	2	28598	2.8	**0.035**
Experimental error A	6	100020			5	79598			6	4516			5	34741		
Comp	2	54637	8.5	**< 0.001**	2	58611	19.4	**< 0.001**	2	6053	37.7	**< 0.001**	2	20792	13.4	**< 0.001**
Prod × Comp	2	18458	2.9	**0.005**	2	12625	4.2	**0.004**	2	429	2.7	0.064	2	6119	4.0	**0.006**
Contrasts *a priori*:																
HPP Soil vs. LPP Soil	1	31604	3.6	**0.002**	1	32023	5.0	**0.002**	1	1756	5.5	**0.003**	1	12209	4.6	**0.006**
HPP Root vs. LPP Root	1	35817	4.0	**0.001**	1	40944	4.8	**0.002**	1	2106	4.4	**0.022**	1	10475	3.0	**0.032**
HPP Rhiz vs. LPP Rhiz	1	40659	4.3	**0.001**	1	36472	5.2	**< 0.001**	1	2356	7.0	**0.003**	1	7315	2.2	0.071
Experimental error B	8	25858			8	12104			8	643			8	6199		
Residual error	194	320890			189	224460			194	12562			189	105050		
Total	214	649830			208	511530			214	33905			208	203350		

df, degrees of freedom; F, pseudo-F value; HPP, high-productivity plots; LPP, low-productivity plots; NPP, null-productivity plots; p, p-value based on 1000 permutations; SS, sum of squares.

Experimental error A, Site(Productivity); Experimental error B, Compartment × Site(Productivity); Prod, productivity; Comp, compartment; Rhiz, rhizosphere. Values in bold indicate statistical significance at *p*< 0.05.

Since the null-productivity level only represents samples from the soil compartment, the interaction term was tested with low- and high-productivity levels only.

The taxonomic profiles based on relative abundance differed less at higher taxonomic levels (**Figure S9A, B**). Regarding the ecological functions, the predicted functional profiles of fungi were more similar between productivity levels than those of bacteria (**Figure S9C, D**). For bacteria, a decreasing trend was found for chemoheterotrophic functions between the HPP, LPP and NPP, while an increasing trend was found for nitrogen cycling. On the other hand, nitrogen fixation alone showed an opposite trend: it was predicted with a relative abundance of 1.74%, 0.42% and 0.65% in the HPP, LPP and NPP, respectively, and it was systematically higher in the HPP than in the LPP for the rhizosphere and root compartments. This trend was supported by the real-time qPCR analysis, which showed that the number of copies of *nifH* per gram of soil or root sample was 6 to 7 times greater in the HPP than in the LPP and NPP (**Figure 2**). Finally, the bacterial and fungal biomass values obtained for the soil and rhizosphere (**Figure 2**) in the HPP were 3 to 6 times higher than in the LPP, and up to 19 times higher in the HPP than in the NPP for soil fungal biomass (*p*< 0.01).

### 3.2 High productivity plots are characterized by microorganisms that specialize in nutrient extraction

**Figure 3:**
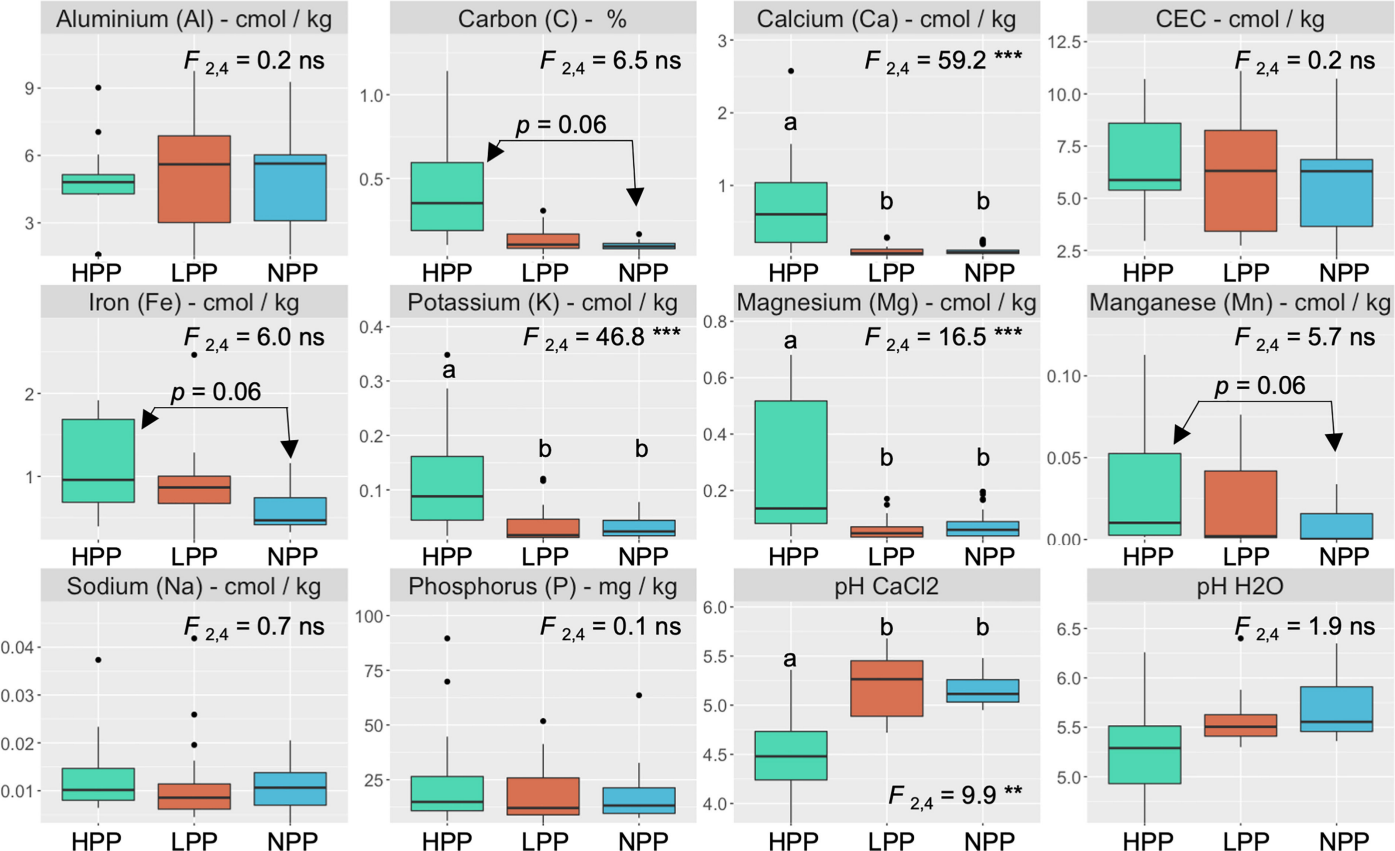
Nutrient concentrations and pH values in the topsoil (0–20 cm) as a function of productivity levels (HPP, high-productivity plots; LPP, low-productivity plots; NPP, null-productivity plots). The *p* value is the result of a Tukey’s multiple comparison test performed on the productivity levels. *F* values are associated with the effect of productivity levels using linear mixed models and symbols: ns, **, *** indicate *p* > 0.05, *p*< 0.01 and *p*< 0.001, respectively.

**Figure 4:**
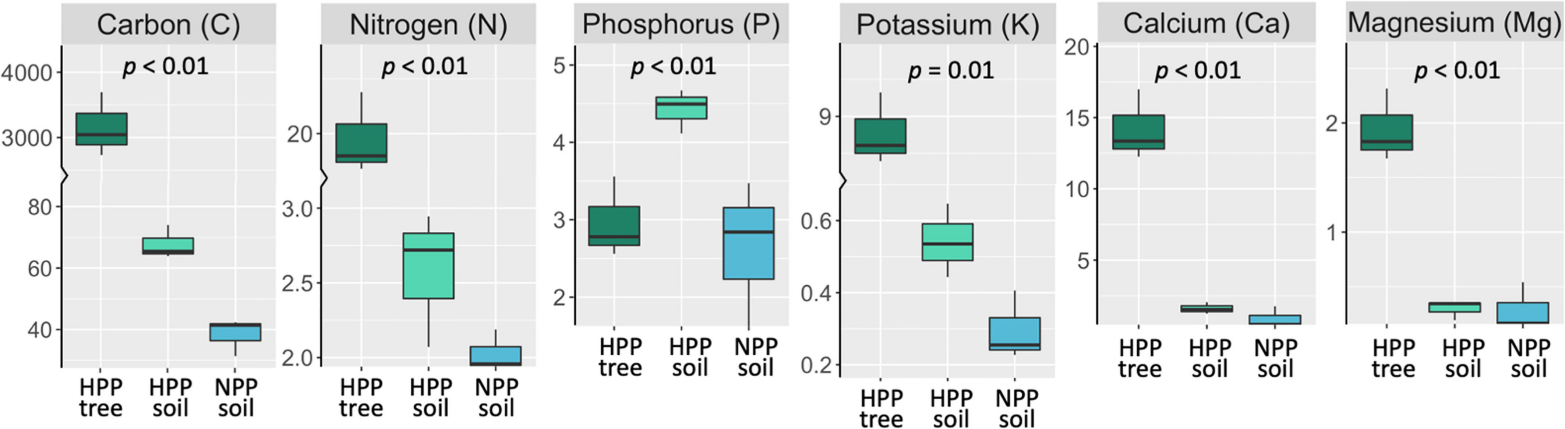
Stocks (g/m^-2^) of total C, total N and extractable P, K, Ca, and Mg measured in tree tissue (dark green) and topsoil (light green) in high-productivity plots (HPP) and in soil only (blue) in null-productivity plots (NPP) where the vegetation biomass was negligible (see **Figures S2B, E**). The comparison of the sum of C and nutrient stocks in vegetation and soil between HPP and NPP gives an approximation of the accumulation of these elements over 16 years of ecosystem development, assuming that the stocks found in NPP represent initial conditions. The *p* value is the result of a t-test comparing nutrient stocks (tree + soil) between HPP and NPP. More details on the estimated nutrient accretion in HPP can be found in **Table 1**.

The microbiome composition in the HPP was associated with higher concentrations of C, Ca, K, and Mg and lower pH values (**Figures 1**, **3**). The median concentration of these elements was about three times higher in the HPP than the LPP and NPP (*p* ≤ 0.06), while the sum of the vegetation and soil nutrient stocks was higher in the HPP than in the NPP (**Figure 4**, **Table 1**). C as well as N, P and K stocks were significantly greater in the HPP than the NPP (*p* ≤ 0.01). In the HPP, the tree tissues stored more C, N, K, and Mg than the soil and were therefore the main reservoir of accumulated carbon and nutrients, except for P. Phosphorus was mainly stored in the soil in the HPP ( $\bar{x}$ = 4.4 g/m^-2^), while a similar amount of P was recorded in tree tissues in the HPP ( $\bar{x}$ = 2.9 g/m^-2^) and soil in the NPP ( $\bar{x}$ = 2.6 g/m^-2^). The average rate of nutrient accretion ranged from 1.2 kg ha^-1^ y^-1^ for Mg to 12.5 kg ha^-1^ y^-1^ for N (**Table 1**) in HPP. Assuming an unlikely 100% retention over the 16 years of the experiment, the RCW amendment would account for 24%, 14%, 42%, 90%, and 56% of the accumulated N, P, K, Ca and Mg, respectively (**Table 1**).

**Figure 5:**
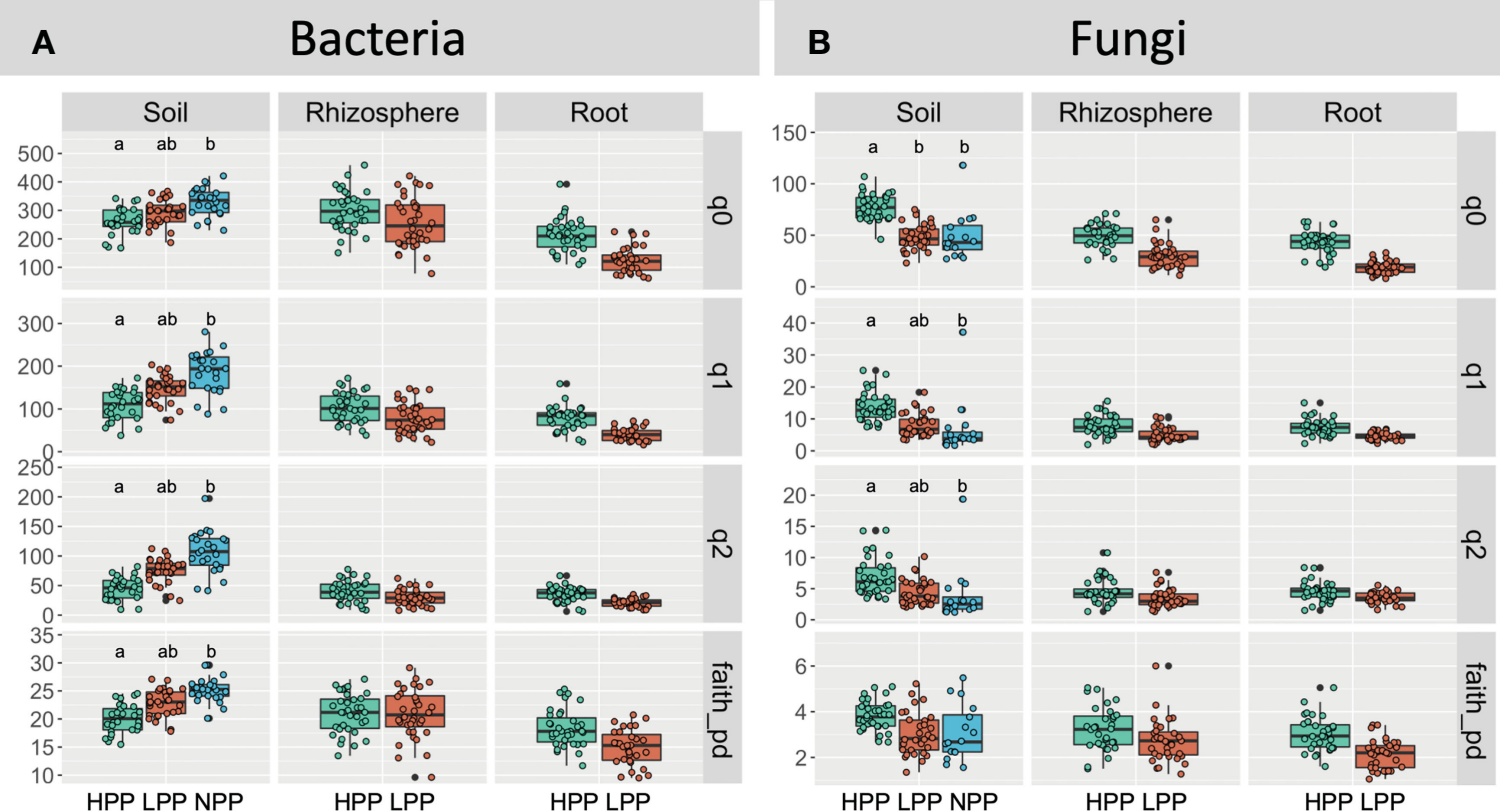
Hill’s diversity calculated for the bacterial **(A)** and fungal **(B)** communities as a function of the productivity levels (HPP, high-productivity plots; LPP, low-productivity plots; NPP, null-productivity plots) and compartments (bulk soil, rhizosphere and roots), species richness (*q* = 0), Shannon diversity (*q* = 1), Simpson diversity (*q* = 2) and Faith’s phylogenetic diversity index. For fungi, Faith’s phylogenetic diversity index was calculated using agaricomycetes only. Medians are indicated by black horizontal lines. Boxplots with a different letters indicate significant differences between treatments (*p*< 0.05). Non-significant differences are not shown.

The levels of forest reclamation and tree productivity had contrasting effects on the alpha diversity of bacteria and of fungi (**Figure 5**). The diversity indices obtained for soil bacteria were greater in the NPP than in the HPP and LPP (*p*< 0.05). For example, the effective number of species derived from Shannon entropy (q = 2) recorded in the soil samples decreased by 20% to 26% from the NPP to the HPP (NPP: 185 ± 49, LPP: 147± 31, HPP: 108± 35). However, this trend was not consistent between soil, rhizosphere, and root samples: the bacterial diversity in root and rhizosphere tended to increase with tree productivity, but the differences were not significant at *p* = 0.05. In contrast to bacterial diversity, the highest values of fungal diversity were recorded in the HPP, and this was consistent between the soil, rhizosphere, and root samples. However, only the species’ richness of the soil fungi was significantly higher (*p*< 0.05) in the HPP (77 ± 15) than in the LPP (48 ± 12) and NPP (45 ± 14).

**Figure 6:**
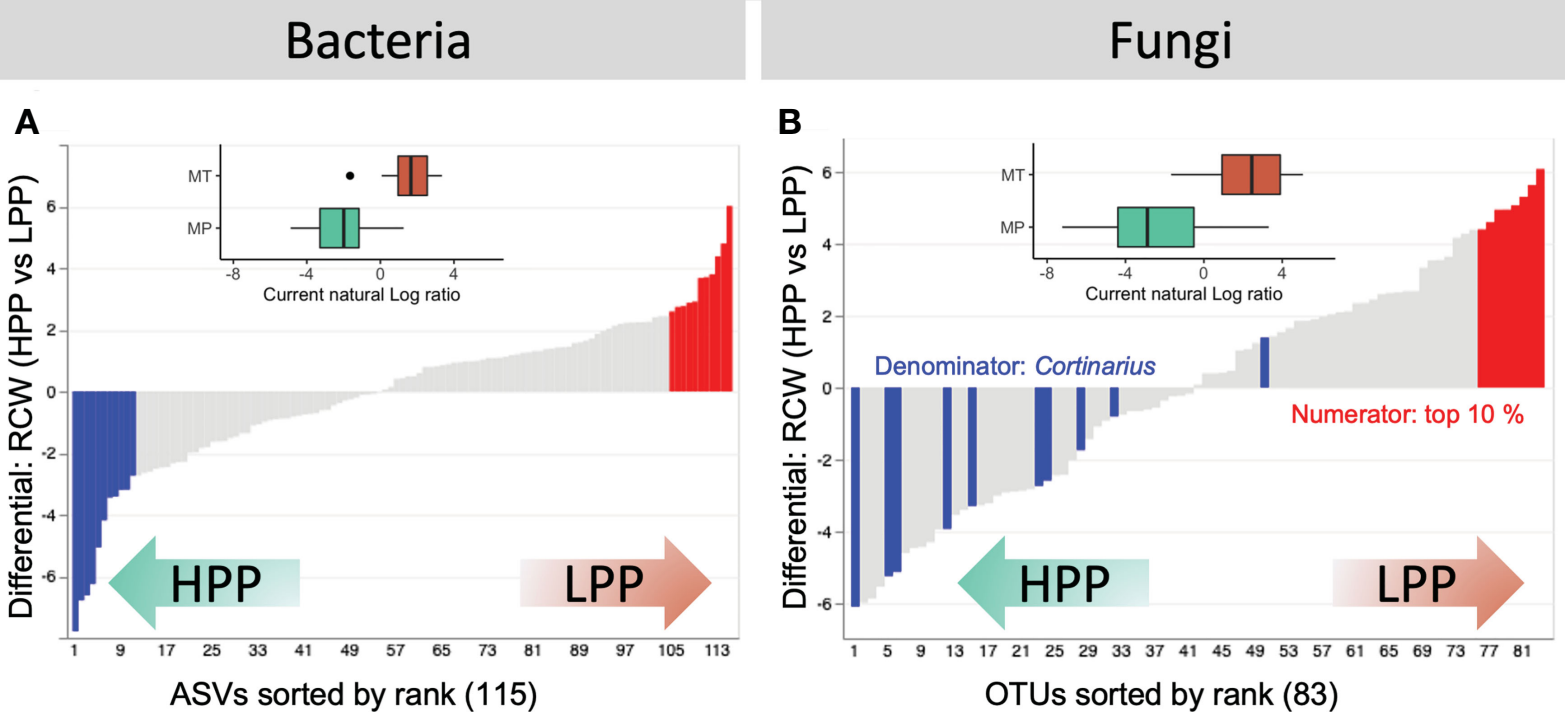
Rank plots showing differentials computed based on association with high-productivity plot (HPP) and low-productivity plot (LPP) samples, using the HPP as the reference category in the regression. **(A)** The top (numerator, red) and bottom (denominator, blue) 10% of the bacterial ASVs were selected. **(B)** For fungal OTUs, the log-ratio of classified *Cortinarius* OTUs to the top 10% of OTUs was selected. Inset boxplots show log-ratios by productivity level (high - HPP and low - LPP). Note that only 66/208 (28.85%) and 126/209 (60.29%) samples are represented in the boxplots for bacteria and fungi, respectively. Other samples were either filtered out prior to the analysis or contained zeros on at least one side of their log-ratio.

Samples from LPP and HPP could be distinguished on the basis of the differentially abundant bacteria and fungi (**Figure 6**). The functional screening of 16S-derived bacterial data was performed using FAPROTAX, which showed that decomposers (*Sinobacteraceae*), heterotrophic ammonia oxidizing bacteria (*Xanthomonadaceae* and *Chitinophagaceae*), and denitrifying bacteria (*Comamonadaceae* and *Actinomycetales*) were significantly more abundant in the HPP (**Table S3**). The LPP provided a favorable niche for taxa related to the *Mycobacteriaceae*, *Koribacteraceae*, and *Sinobacteraceae* and as well as for *Chitinophagaceae* and *Burkholderiaceae*. Ectomycorrhizal fungi related to the genus *Cortinarius* dominated the top contributing OTUs in the HPP (**Figure 6B**, **Table S4**). The log-ratio of *Cortinarius* OTUs classified in the top 10% of OTUs showed a clear separation between the LPP and HPP (inset boxplot, **Figure 6B**, **Table S5**), indicating that *Cortinarius* taxa were key players in the HPP, both in terms of diversity and abundance.

### 3.3 Network inference of underground microorganisms

**Figure 7:**
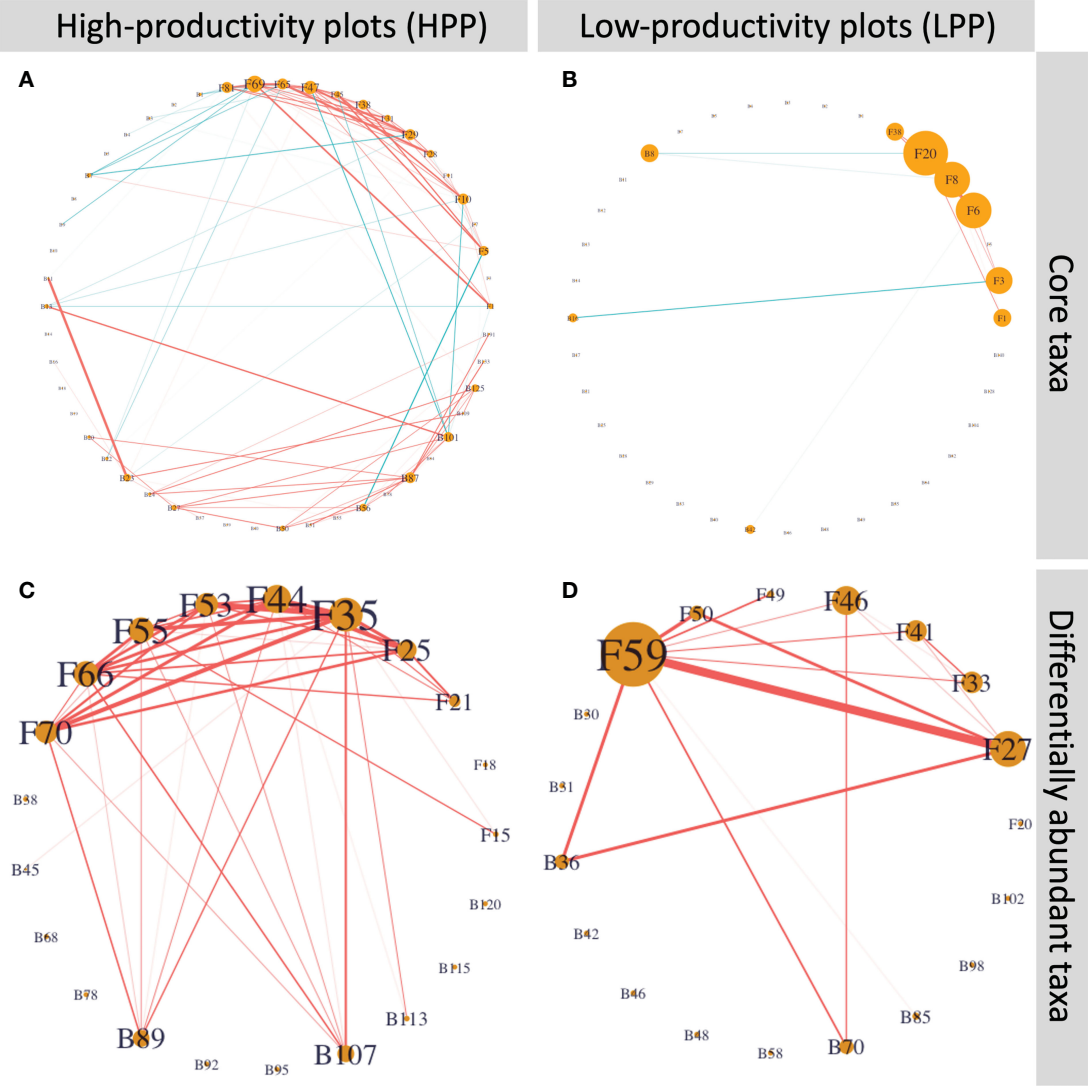
**(A)** and **(B)** Inferred networks for the core taxa and **(C)** and **(D)** the differentially abundant taxa. Network nodes labeled with “B” and “F” represent bacterial ASVs and fungal OTUs, respectively. Edge thickness is proportional to the node associations and significant positive or negative associations are indicated by the color red or blue, respectively. Node size is proportional to degree, which is the number of edges connecting each node to the rest of the network.**Table S6** shows the taxonomic identification for each node and its number of interactions.

The core and differentially abundant taxa had a low connectivity in the LPP (**Figure 7**). In the HPP, the network based on the core microbiome had approximately 10 times more edges (110 versus 12) than the network in the LPP. For the differentially abundant taxa, the number of edges was twice as high as in the HPP (42 edges) than the LPP (21 edges). Nine out of ten *Cortinarius* species were interconnected and two bacterial taxa related to the *Chitinophagaceae* were positively associated with six out of ten *Cortinarius* species. In the LPP, *Lactarius sp*. (OTU059) and *Rhizopogon pseudoproseolus* (OTU027) were most associated with the other differentially abundant fungi and bacteria. Two bacterial taxa related to the *Sinobacteraceae* (ASV0070) and the genus *Burkholderia* (ASV0036) were positively associated with *Lactarius* sp. (OTU059), *Rhizopogon pseudoproseolus* (OTU027) and *Tomentella* sp. (OTU046).

## 4 Discussion

Successful reforestation of borrow pits in boreal forests requires the establishment of environmental conditions that can initiate a positive PSF. Planting trees on a continuous blanket of RCW has allowed the restoration of a forest ecosystem and initiated a positive PSF. In fact, the differences between RCW inputs and total nutrient accumulation indicate a higher level of nutrient accretion and nutrient accumulation in the HPP. This supports a positive PSF involving a specialized soil microbiome that is more efficient at nutrient mobilization and acquisition. The in-depth characterization of the belowground microbiome and nutrient stocks and concentrations in plots that exhibit a gradient of forest restoration success and productivity, 17 years after the field trial began, show how the topsoil microbiome and biochemistry contribute to positive PSF and recovery of soil health.

### 4.1 High mineral weathering and non-symbiotic N fixation support positive PSF

The contrasting levels of productivity observed in each experimental unit demonstrated the importance of applying RCW as a continuous layer over plots to be reforested in borrow pits. The wood mulch likely provided protection from the extreme abiotic conditions and its decomposition provided the nutrients necessary for seedling establishment. This allowed the seedlings to promote enhanced mineral weathering of the coarse sand particles found in the borrow pits through the recruitment of a specialized soil microbiome. Ectomycorrhizal fungi (van Schöll et al., 2008), bacteria (Uroz et al., 2009) and their interactions (Koele et al., 2009; Fontaine et al., 2016) contribute to accelerated mineral weathering rates. Their activity supported the development of trees measuring up to 6 m tall, 17 years after reclamation of the sand borrow pits began. The Hubbard Brook sandbox experiment showed that unvegetated soils had negligible weathering, while mineral weathering for K, Ca and Mg was high on vegetated plots (Bormann et al., 1998). The rate of mineral weathering required for ecosystem nutrient accretion appears to be high in the HPP compared to the long-term mineral weathering rates in granitic soils observed by Vadeboncoeur et al. (2014). For example, these authors reported Ca rates ranging from 0.1 to 4 kg ha^-1^ y^-1^, whereas Ca accretion rates in the HPP were approximately 9 kg ha^-1^ y^-1^ (**Table 1**). Our results show significant nutrient inputs from mineral weathering in the upper portion of the soil, even after accounting for inputs from wood mulch and Ca in precipitation (which are likely less than 1 kg ha^-1^ y^-1^, Vadeboncoeur et al., 2014). In addition, soil acidification in the HPP, which was likely due to EM fungi, which accounted for the highest fungal biomass (Bahram et al., 2020), and root exudates and decaying soil organic matter, which released organic acids (Jones, 1998), also contributed to mineral weathering.

With respect to nitrogen, the ecosystem rate of N accretion in the HPP (12.5 kg ha^-1^ y^-1^) was six to twelve times higher than the estimated atmospheric N deposition rate for this region (1 and 2 kg ha^-1^ y^-1^ according to the Oak Ridge National Laboratory [ORNL] Distributed Active Archive Center [DAAC], data from July 15, 2016). The N inputs through the fertilizer applied at planting (**Table S1**) in addition to that contained in the wood mulch (**Table 1**) amount to at most to 50% of the N accretion, assuming an unrealistic level of 100% fertilizer retention in the HPP and noting that fertilizer was also applied to all planted trees in all treatments. Therefore, some of the N accretion in the HPP was likely the product of non-symbiotic N fixation (Johnson and Turner, 2014). For example, N-fixing activity has been associated with the ectomycorrhizal fungi *Suillus variegatus, S. tomentosus* and *Tomentellopsis submollis* on pine roots (Izumi et al., 2006; Paul et al., 2007). Our results showed that *Suillus tomentosus* (OTU007) and *S. grevillei* (OTU029) were part of the core fungal community in the HPP (**Figure S8**, **Table S8**). Similarly, families of free-living and plant symbiotic nitrogen fixers (*Bradyrhizobiaceae*, *Burkholderiaceae*, *Methylocystaceae*, *Rhizobiaceae*) were dominant in the HPP. The genera *Bradyrhizobium* and *Burkholderia* are generally the most abundant in soil microbiota (Shah and Subramaniam, 2018), and ASVs assigned to these genera dominated the core bacterial community in each plot. However, the number of copies of the *nifH* gene indicated that taxa from these groups thrived only in the HPP. EM fungi can utilize both inorganic and organic nitrogen, and efficiently immobilize nitrogen and redistribute it to their hosts through common mycorrhizal networks (He et al., 2003; Sterkenburg et al., 2018). In addition, EM fungi likely received more carbohydrates from the vigorous jack pines than from the stunted pines, which would increase mycelium biomass and nitrogen retention in mycelium (Näsholm et al., 2013).

Although the *post hoc* nature of the current study prevents a precise assessment of nutrient inputs (lack of information on soil and wood mulch conditions at time zero), our estimates of external nutrient sources including those from wood mulch and atmospheric deposition suggest that PSF contributes to the nutrient accumulation observed in HPP. The environmental conditions at our sites, with an extremely poor soils and a boreal climate are also conducive to PSF. In fact, PSF is more commonly reported for extreme environments (Ehrenfeld et al., 2005).

### 4.2 Positive feedback is microbially mediated

More fungi-fungi, bacteria-bacteria and fungi-bacteria interactions were observed in the HPP than the LPP. The soil microbiome found in the HPP was dominated by *Cortinarius* taxa and *Chitinophagaceae*. *Cortinarius* is the largest genus of ectomycorrhizal fungi (> 2,000 species) (Høiland and Holst-Jensen, 2000; Soop and Gasparini, 2011), and represents approximately 10% of the species forming ectomycorrhizae (Rinaldi et al., 2008). *Cortinarius* species often dominate the ectomycorrhizosphere in reforested plots (Stefani et al., 2009; Pickles et al., 2010; Villarreal-Ruiz and Neri-Luna, 2017; Haas et al., 2018). Their extraradical mycelium is classified as a medium-distance exploratory type (Agerer, 2001; Agerer and Raidl, 2004), in the fringe subtype. This particular type of mycelium forms fans and rhizomorphs that ramify and interconnect repeatedly (Agerer, 2001). Furthermore, species within *Cortinarius* are considered to be nitrophobic taxa (Lilleskov et al., 2001; Lilleskov et al., 2002; Haas et al., 2018). Thus, the oligotrophic soil environment in borrow pits provides suitable conditions for the proliferation of *Cortinarius*. Interestingly, the roots of stunted pines in the LPP were colonized by several EM fungi and were showed a positive correlation with bacterial taxa different from those observed in the HPP. No fungal or bacterial pathogens were detected; therefore, they are unlikely to be responsible for the stunted trees that survived in the LPP. EM fungi were detected even in the absence of host trees in the NPP, indicating the constant presence of substantial inoculum in the surrounding area. Sampling was performed in late autumn, and the phenological timing of the *Basidiomycota* fruiting bodies in late summer may have influenced the spore load in bare soil samples, through wind transport and deposition (Després et al., 2012; Manninen et al., 2014).

The core taxa and the differentially abundant taxa suggest the presence of abundant resources in the HPP. For example, two bacterial taxa from the family *Chitinophagaceae* were significantly more abundant in the HPP and positively correlated with the abundance of *Cortinarius*. Some species in the family *Chitinophagaceae* are predicted to have β-glucosidase activity and are responsible for degrading polymeric organic matter including chitin and cellulose (Bailey et al., 2013). The inferred network for the core taxa showed that ASV0101 (*Acidisoma* sp.), ASV0087 (candidate alphaproteobacterial order Ellin329), OTU069 (*Saccharomycetales*), and OTU047 (*Mortierella*) were putative keystone taxa in the HPP. Sugars, polyalcohols and some organic acids are the preferred growth substrates for taxa within the genus *Acidisoma* (Belova et al., 2009). The presence of fungal yeasts (OTU069) reflects the availability of simple compounds such as sugars (monomers and oligomers), released by the hydrolytic enzyme activities of fungal decomposers or by root exudation. The decomposition of RCW and leaf litter releases polysaccharides and lignin which are likely to stimulate the development of these keystone species. The soil environment in the HPP was also more favorable for chemoheterotrophic bacterial taxa, that derive energy from decomposition of soil organic matter. This is consistent with the highest bacterial biomass values found in the HPP. The bacterial and fungal biomass recorded in these plots reflects nutrient assimilation and active biogeochemical transformation (Mason-Jones et al., 2022).

## 5 Conclusions

Analysis of soil nutrients and the microbiome, performed in a unique long-term reforestation experiment in the boreal forest, showed that the application of RCW at the plot level can provide the conditions necessary for sustainable seedling growth and the initiation of a positive PSF in barren soils under harsh environmental conditions. In plots with the highest load of RCW, seedlings were able to recruit bacterial and fungal taxa that actively enhanced the availability of nutrients produced by mineral weathering and non-symbiotic N fixation. Applying wood mulch in plots rather than around individual trees initiated a microbially mediated PSF that helped transform unproductive plots into productive plots and to ensure rapid restoration of forest ecosystems in a harsh boreal environment.

## Data availability statement

The datasets presented in this study can be found in online repositories. The names of the repository/repositories and accession number(s) can be found below: https://www.ncbi.nlm.nih.gov/sra/, PRJNA775865.

## Author contributions

FS coordinated the study, analyzed the metabarcoding data, performed the differential abundance and network analyses and wrote the manuscript. JB performed all multilevel and multivariate statistical analyses. DP conceived the experimental design. DP and NT planned and supervised the soil and vegetation sampling and analysis. DP contributed to the interpretation of the geochemical nutrient accumulation. MM performed the DNA extraction, amplification and library preparation for Illumina sequencing. CM planned and supervised the qPCR analyses and contributed to microbiome data interpretation. DP, JAF, NT and AS participated in the design of the study. JB, DP, CM, NT and AS contributed to the writing of the manuscript. FS and DP wrote the revised version of the manuscript. All authors contributed to the article and approved the submitted version.

## References

[B1] Agerer R. (2001). Exploration types of ectomycorrhizae - a proposal to classify ectomycorrhizal mycelial systems according to their patterns of differentiation and putative ecological importance. Mycorrhiza 11, 107–114. doi: 10.1007/s005720100108

[B2] Agerer R. Raidl S. (2004). Distance-related semi-quantitative estimation of the extramatrical ectomycorrhizal mycelia of *Cortinarius obtusus* and *Tylospora asterophora* . Mycol. Prog. 3, 57–64. doi: 10.1007/s11557-006-0077-9

[B3] Allaire J. Xie Y. McPherson J. Luraschi J. Ushey K. Atkins A.. (2022) Rmarkdown: Dynamic documents for r. Available at: https://github.com/rstudio/rmarkdown.

[B4] Anderson M. J. Gorley R. N. Clarke K. R. (2008). PERMANOVA+ for PRIMER: guide to software and statistical methods (Plymouth, UK: PRIMER-E). Available at: www.primer-e.com.

[B5] Angel R. Nepel M. Panhölzl C. Schmidt H. Herbold C. W. Eichorst S. A.. (2018). Evaluation of primers targeting the diazotroph functional gene and development of NifMAP – a bioinformatics pipeline for analyzing nifH amplicon data. Front. Microbiol. 9. doi: 10.3389/fmicb.2018.00703 PMC5936773 29760683

[B6] Anslan S. Bahram M. Tedersoo L. (2016). Temporal changes in fungal communities associated with guts and appendages of collembola as based on culturing and high-throughput sequencing. Soil Biol. Biochem. 96, 152–159. doi: 10.1016/j.soilbio.2016.02.006

[B7] Arnold J. (2021) Ggthemes: Extra themes, scales and geoms for ‘ggplot2’. r package version 4.2.4. Available at: https://CRAN.R-project.org/package=ggthemes.

[B8] Auguie B. (2017) gridExtra: Miscellaneous functions for “Grid” graphics. r package version 2.3. Available at: https://CRAN.R-project.org/package=gridExtra.

[B9] Bahram M. Netherway T. Hildebrand F. Pritsch K. Drenkhan R. Loit K.. (2020). Plant nutrient-acquisition strategies drive topsoil microbiome structure and function. New Phytol. 227, 1189–1199. doi: 10.1111/nph.16598 32279325

[B10] Bailey V. L. Fansler S. J. Stegen J. C. McCue L. A. (2013). Linking microbial community structure to β-glucosidic function in soil aggregates. Isme J. 7, 2044–2053. doi: 10.1038/ismej.2013.87 23719152 PMC3965315

[B11] Bates D. Mächler M. Bolker B. Walker S. (2015). Fitting linear mixed-effects models using lme4. J. Stat. Softw. 67, 1–48. doi: 10.18637/jss.v067.i01

[B12] Belova S. E. Pankratov T. A. Detkova E. N. Kaparullina E. N. Dedysh S. N. (2009). *Acidisoma tundrae* gen. nov., sp. nov. and *Acidisoma sibiricum* sp. nov., two acidophilic, psychrotolerant members of the alphaproteobacteria from acidic northern wetlands. Int. J. Syst. Evol. Micr. 59, 2283–2290. doi: 10.1099/ijs.0.009209-0 19620354

[B13] Bennett J. A. Maherali H. Reinhart K. O. Lekberg Y. Hart M. M. Klironomos J. (2017). Plant-soil feedbacks and mycorrhizal type influence temperate forest population dynamics. Science 355, 181–184. doi: 10.1126/science.aai8212 28082590

[B14] Bergmann J. Verbruggen E. Heinze J. Xiang D. Chen B. Joshi J.. (2016). The interplay between soil structure, roots, and microbiota as a determinant of plant–soil feedback. Ecol. Evol. 6, 7633–7644. doi: 10.1002/ece3.2456 30128117 PMC6093149

[B15] Bever J. D. Westover K. M. Antonovics J. (1997). Incorporating the soil community into plant population dynamics: the utility of the feedback approach. J. Ecol. 85, 561. doi: 10.2307/2960528

[B16] Bolyen E. Rideout J. Dillon M. R. Bokulich N. A. Abnet C. C. Al-Ghalith G. A.. (2019). Reproducible, interactive, scalable and extensible microbiome data science using QIIME 2. Nat. Biotechnol. 37, 848–857. doi: 10.1038/s41587-019-0209-9 31341288 PMC7015180

[B17] Bormann B. T. Wang D. Snyder M. C. Bormann F. H. Benoit G. April R. (1998). Rapid, plant-induced weathering in an aggrading experimental ecosystem. Biogeochemistry 43, 129–155. doi: 10.1023/a:1006065620344

[B18] Callahan B. J. McMurdie P. J. Rosen M. J. Han A. W. Johnson A. A. Holmes S. P. (2016). DADA2: High-resolution sample inference from illumina amplicon data. Nat. Methods. Boca Raton 13, nmeth.3869. doi: 10.1038/nmeth.3869 PMC4927377 27214047

[B19] Carter M. R. Gregorich E. G. (Eds.) (2007). Soil sampling and methods of analysis. 2nd ed (Boca Raton: CRC Press). doi: 10.1201/9781420005271

[B20] Chao A. Gotelli N. J. Hsieh T. Sander E. L. Ma K. Colwell R. K.. (2014). Rarefaction and extrapolation with hill numbers: a framework for sampling and estimation in species diversity studies. Ecol. Monogr. 84, 45–67. doi: 10.1890/13-0133.1

[B21] Chazdon R. L. (2008). Beyond deforestation: restoring forests and ecosystem services on degraded lands. Science 320, 1458–1460. doi: 10.1126/science.1155365 18556551

[B22] Chiquet J. Mariadassou M. Robin S. (2018). Variational inference for probabilistic poisson PCA. Ann. Appl. Stat 12, 2674–2698. doi: 10.1214/18-AOAS1177.full

[B23] Chiquet J. Mariadassou M. Robin S. (2019a). “Variational inference for sparse network reconstruction from count data,” in Proceedings of the 36th International Conference on Machine Learning Proceedings of the 36th International Conference on Machine Learning (ICML), Long Beach, California, 1162–1171.

[B24] Chiquet J. Mariadassou M. Robin S. (2019b) Variational inference for sparse network reconstruction from count data. Available at: http://proceedings.mlr.press/v97/chiquet19a.html.

[B25] Chiquet J. Mariadassou M. Robin S. (2021). The poisson-lognormal model as a versatile framework for the joint analysis of species abundances. Front. Ecol. Evol. 9. doi: 10.3389/fevo.2021.588292

[B26] Csardi G. Nepusz T. (2006) The igraph software package for complex network research, InterJournal, complex systems 1695. Available at: https://igraph.org.

[B27] de Souza Y. A. F. Leite M. G. P. Fujaco M. A. G. (2021). A hydroelectric dam borrow pit rehabilitation. two decades after the project, what went wrong? J. Environ. Manage 293, 112850. doi: 10.1016/j.jenvman.2021.112850 34052612

[B28] Després V. R. Huffman J. A. Burrows S. M. Hoose C. Safatov A. S. Buryak G.. (2012). Primary biological aerosol particles in the atmosphere: a review. Tellus B 64, 15598. doi: 10.3402/tellusb.v64i0.15598

[B29] Ehrenfeld J. G. Ravit B. Elgersma K. (2005). Feedback in the plant-soil system. Annu. Rev. Env. Resour. 30, 75–115. doi: 10.1146/annurev.energy.30.050504.144212

[B30] Faith D. P. Baker A. M. (2006). Phylogenetic diversity (PD) and biodiversity conservation: some bioinformatics challenges. Evol. Bioinform. 2, 121–128. doi: 10.1177/117693430600200007 PMC2674678 19455206

[B31] Fedarko M. W. Martino C. Morton J. T. González A. Rahman G. Marotz C. A.. (2020). Visualizing’ omic feature rankings and log-ratios using qurro. Nar Genom. Bioinform. 2, lqaa023. doi: 10.1093/nargab/lqaa023 32391521 PMC7194218

[B32] Fontaine L. Thiffault N. Paré D. Fortin J.-A. Piché Y. (2016). Phosphate-solubilizing bacteria isolated from ectomycorrhizal mycelium of *Picea glauca* are highly efficient at fluorapatite weathering. Botany 94, 1183–1193. doi: 10.1139/cjb-2016-0089

[B33] Høiland K. Holst-Jensen A. (2000). *Cortinarius* phylogeny and possible taxonomic implications of ITS rDNA sequences. Mycologia. 92, 694–710. doi: 10.1080/00275514.2000.12061210

[B34] Haas J. C. Street N. R. Sjödin A. Lee N. M. Högberg M. N. Näsholm T.. (2018). Microbial community response to growing season and plant nutrient optimisation in a boreal Norway spruce forest. Soil Biol. Biochem. 125, 197–209. doi: 10.1016/j.soilbio.2018.07.005

[B35] Habiyaremye J. de D. Goldmann K. Reitz T. Herrmann S. Buscot F. (2020). Tree root zone microbiome: exploring the magnitude of environmental conditions and host tree impact. Front. Microbiol. 11. doi: 10.3389/fmicb.2020.00749 PMC7190799 32390986

[B36] He X.-H. Critchley C. Bledsoe C. (2003). Nitrogen transfer within and between plants through common mycorrhizal networks (CMNs). Crit. Rev. Plant Sci. 22, 531–567. doi: 10.1080/713608315

[B37] Hill M. O. (1973). Diversity and evenness: a unifying notation and its consequences. Ecology 54, 427–432. doi: 10.2307/1934352

[B38] Horton T. R. (2017). “Spore dispersal in ectomycorrhizal fungi at fine and regional scales,” in Ecological studies (Cham: Springer International Publishing), 61–78. doi: 10.1007/978-3-319-56363-3_3

[B39] Hothorn T. Bretz F. Westfall P. (2008). Simultaneous inference in general parametric models. Biometric. J. 50, 346–363. doi: 10.1002/bimj.200810425 18481363

[B40] Hsieh T. C. Ma K. H. Chao A. (2016). iNEXT: an r package for rarefaction and extrapolation of species diversity (Hill numbers). Methods Ecol. Evol. 7, 1451–1456. doi: 10.1111/2041-210x.12613

[B41] Hugron S. Andersen R. Poulin M. Rochefort L. (2011). Natural plant colonization of borrow pits in boreal forest highlands of eastern Canada. Botany 89, 451–465. doi: 10.1139/b11-036

[B42] Izumi H. Anderson I. C. Alexander I. J. Killham K. Moore E. R. B. (2006). Diversity and expression of nitrogenase genes (nifH) from ectomycorrhizas of Corsican pine (*Pinus nigra*). Environ. Microbiol. 8, 2224–2230. doi: 10.1111/j.1462-2920.2006.01104.x 17107563

[B43] Johnson D. W. Turner J. (2014). Nitrogen budgets of forest ecosystems: A review. For. Ecol. Manage. 318, 370–379. doi: 10.1016/j.foreco.2013.08.028

[B44] Jones D. L. (1998). Organic acids in the rhizosphere – a critical review. Plant Soil 205, 25–44. doi: 10.1023/a:1004356007312

[B45] Karlsson E. Johansson A.-M. Ahlinder J. Lundkvist M. J. Singh N. J. Brodin T.. (2020). Airborne microbial biodiversity and seasonality in northern and southern Sweden. Peerj 8, e8424. doi: 10.7717/peerj.8424 32025374 PMC6991134

[B46] Katoh K. Misawa K. Kuma K. Miyata T. (2002). MAFFT: a novel method for rapid multiple sequence alignment based on fast Fourier transform. Nucleic Acids Res. 30, 3059–3066. doi: 10.1093/nar/gkf436 12136088 PMC135756

[B47] Koele N. Turpault M.-P. Hildebrand E. E. Uroz S. Frey-Klett P. (2009). Interactions between mycorrhizal fungi and mycorrhizosphere bacteria during mineral weathering: Budget analysis and bacterial quantification. Soil Biol. Biochem. 41, 1935–1942. doi: 10.1016/j.soilbio.2009.06.017

[B48] Kulmatiski A. Beard K. H. Stevens J. R. Cobbold S. M. (2008). Plant–soil feedbacks: a meta-analytical review. Ecol. Lett. 11, 980–992. doi: 10.1111/j.1461-0248.2008.01209.x 18522641

[B49] Lahti L. Shetty S. (2019) Microbiome r package. Available at: http://microbiome.github.io/.

[B50] Lenth R. V. (2016). Least-squares means: The r package *lsmeans* . J. Stat. Softw. 69, 1–33. doi: 10.18637/jss.v069.i01

[B51] Li C. Y. Hung L. L. (1987). Nitrogen-fixing (acetylene-reducing) bacteria associated with ectomycorrhizae of Douglas-fir. Plant Soil 98, 425–428. doi: 10.1007/bf02378363

[B52] Lilleskov E. A. Bruns T. D. (2005). Spore dispersal of a resupinate ectomycorrhizal fungus, *Tomentella sublilacina*, *via* soil food webs. Mycologia 97, 762–769. doi: 10.1080/15572536.2006.11832767 16457345

[B53] Lilleskov E. Fahey T. Horton T. Lovett G. (2002). Belowground ectomycorrhizal fungal community change over a nitrogen deposition gradient in Alaska. Ecology 83, 104–115. doi: 10.1890/0012-9658(2002)083[0104:BEFCCO]2.0.CO;2

[B54] Lilleskov E. A. Fahey T. J. Lovett G. M. (2001). Ectomycorrhizal fungal aboveground community change over an atmospheric nitrogen deposition gradient. Ecol. Appl. 11, 397–410. doi: 10.1890/1051-0761(2001)011[0397:EFACCO]2.0.CO;2

[B55] Liu C. M. Kachur S. Dwan M. G. Abraham A. G. Aziz M. Hsueh P.-R.. (2012). FungiQuant: A broad-coverage fungal quantitative real-time PCR assay. BMC Microbiol. 12, 255. doi: 10.1186/1471-2180-12-255 23136846 PMC3565980

[B56] Louca S. Parfrey L. W. Doebeli M. (2016). Decoupling function and taxonomy in the global ocean microbiome. Science 353, 1272–1277. doi: 10.1126/science.aaf4507 27634532

[B57] Macdonald S. E. Landhäusser S. M. Skousen J. (2015). Forest restoration following surface mining disturbance: challenges and solutions. New Forests 46, 703–732. doi: 10.1007/s11056-015-9506-4

[B58] Manninen H. E. Bäck J. Sihto-Nissilä S.-L. Huffman J. A. Pessi A.-M. Hiltunen V.. (2014). Patterns in airborne pollen and other primary biological aerosol particles (PBaP), and their contribution to aerosol mass and number in a boreal forest. Boreal Environ. Res. 19, 383–405.

[B59] Mason-Jones K. Robinson S. L. Veen (Ciska) G. F. Manzoni S. van der Putten W. H. (2022). Microbial storage and its implications for soil ecology. Isme J. 16, 617–629. doi: 10.1038/s41396-021-01110-w 34593996 PMC8857262

[B60] McDonald D. Price M. N. Goodrich J. Nawrocki E. P. DeSantis T. Z. Probst A.. (2012). An improved greengenes taxonomy with explicit ranks for ecological and evolutionary analyses of bacteria and archaea. ISME J. 6, 610–618. doi: 10.1038/ismej.2011.139 22134646 PMC3280142

[B61] McMurdie P. J. Holmes S. (2013). Phyloseq: An r package for reproducible interactive analysis and graphics of microbiome census data. PloS One 8, e61217. doi: 10.1371/journal.pone.0061217.s002 23630581 PMC3632530

[B62] Menkis A. Burokienė D. Gaitnieks T. Uotila A. Johannesson H. Rosling A.. (2012). Occurrence and impact of the root-rot biocontrol agent *Phlebiopsis gigantea* on soil fungal communities in *Picea abies* forests of northern Europe. FEMS Microbiol. Ecol. 81 (2), 438–445. doi: 10.1111/j.1574-6941.2012.01366.x 22443512

[B63] Miller R. O. (1997). “High-temperature oxidation: dry ashing,” in Handbook of reference methods for plant analysis, vol. 1988 . Ed. Kaira Y. P. (Boca Raton: CRC), 53–56.

[B64] Morton J. T. Marotz C. Washburne A. Silverman J. Zaramela L. S. Edlund A.. (2019). Establishing microbial composition measurement standards with reference frames. Nat. Commun. 10, 2719. doi: 10.1038/s41467-019-10656-5 31222023 PMC6586903

[B65] Näsholm T. Högberg P. Franklin O. Metcalfe D. Keel S. G. Campbell C.. (2013). Are ectomycorrhizal fungi alleviating or aggravating nitrogen limitation of tree growth in boreal forests? New Phytol. 198, 214–221. doi: 10.1111/nph.12139 23356503

[B66] Nguyen N. H. Song Z. Bates S. T. Branco S. Tedersoo L. Menke J.. (2016). FUNGuild: An open annotation tool for parsing fungal community datasets by ecological guild. Fungal Ecol. 20, 241–248. doi: 10.1016/j.funeco.2015.06.006

[B67] Nilsson R. H. Larsson K.-H. H. Taylor A. F. S. F. Bengtsson-Palme J. Jeppesen T. S. Schigel D.. (2019). The UNITE database for molecular identification of fungi: handling dark taxa and parallel taxonomic classifications. Nucleic Acids Res. 47, D259–D264. doi: 10.1093/nar/gky1022 30371820 PMC6324048

[B68] Okubo T. Liu D. Tsurumaru H. Ikeda S. Asakawa S. Tokida T.. (2015). Elevated atmospheric CO_2_ levels affect community structure of rice root-associated bacteria. Front. Microbiol. 6. doi: 10.3389/fmicb.2015.00136 PMC4335179 25750640

[B69] Parada A. E. Needham D. M. Fuhrman J. A. (2016). Every base matters: assessing small subunit rRNA primers for marine microbiomes with mock communities, time series and global field samples. Environ. Microbiol. 18, 1403–1414. doi: 10.1111/1462-2920.13023 26271760

[B70] Paul L. R. Chapman B. K. Chanway C. P. (2007). Nitrogen fixation associated with *Suillus tomentosus* tuberculate ectomycorrhizae on *Pinus contorta* var. *latifolia* . Ann. Bot-london 99, 1101–1109. doi: 10.1093/aob/mcm061 PMC3243579 17468111

[B71] Pickles B. J. Genney D. R. Potts J. M. Lennon J. J. Anderson I. C. Alexander I. J. (2010). Spatial and temporal ecology of scots pine ectomycorrhizas. New Phytol. 186, 755–768. doi: 10.1111/j.1469-8137.2010.03204.x 20202132

[B72] Pinheiro J. Bates D. R Core Team (2020) Nlme: Linear and nonlinear mixed effects models. r package version 3.1-152. Available at: https://CRAN.R-project.org/package=nlme.

[B73] Plasse C. Payette S. (2015). Frost hollows of the boreal forest: a spatiotemporal perspective. J. Ecol. 103, 669–678. doi: 10.1111/1365-2745.12399

[B74] Price M. N. Dehal P. S. Arkin A. P. (2010). FastTree 2 – approximately maximum-likelihood trees for large alignments. PloS One 5, e9490. doi: 10.1371/journal.pone.0009490 20224823 PMC2835736

[B75] Pritsch K. Garbaye J. (2011). Enzyme secretion by ECM fungi and exploitation of mineral nutrients from soil organic matter. Ann. For. Sci. 68, 25–32. doi: 10.1007/s13595-010-0004-8

[B76] Puri A. Padda K. P. Chanway C. P. (2020). Can naturally-occurring endophytic nitrogen-fixing bacteria of hybrid white spruce sustain boreal forest tree growth on extremely nutrient-poor soils? Soil Biol. Biochem. 140, 107642. doi: 10.1016/j.soilbio.2019.107642

[B77] R Core Team (2020). R: A language and environment for statistical computing (Vienna, Austria: R Foundation for Statistical Computing). Available at: https://www.R-project.org.

[B78] Reynolds H. L. Packer A. Bever J. D. Clay K. (2003). Grassroots ecology: plant–microbe–soil interactions as drivers of plant community structure and dynamics. Ecology 84, 2281–2291. doi: 10.1890/02-0298

[B79] Rinaldi A. C. Comandini O. Kuyper T. W. (2008). Ectomycorrhizal fungal diversity: separating the wheat from the chaff. Fungal Diversity 33, 1–45.

[B80] Rognes T. Flouri T. Nichols B. Quince C. Mahé F. (2016). VSEARCH: a versatile open source tool for metagenomics. PeerJ 4, 1–22. doi: 10.7717/peerj.2584 PMC5075697 27781170

[B81] Saleem M. Hu J. Jousset A. (2019). More than the sum of its parts: microbiome biodiversity as a driver of plant growth and soil health. Annu. Rev. Ecol. Evol. Syst. 50, 1–24. doi: 10.1146/annurev-ecolsys-110617-062605

[B82] Shah V. Subramaniam S. (2018). *Bradyrhizobium japonicum* USDA110: a representative model organism for studying the impact of pollutants on soil microbiota. Sci. Total Environ. 624, 963–967. doi: 10.1016/j.scitotenv.2017.12.185 29275259

[B83] Smits M. M. Bonneville S. Haward S. Leake J. R. (2008). Ectomycorrhizal weathering, a matter of scale? Mineral Mag 72, 131–134. doi: 10.1180/minmag.2008.072.1.131

[B84] Soop K. Gasparini B. (2011). Europe And the south pacific: A comparison of two *Cortinarius* floras. J. Des. JEC 14, 34–45.

[B85] Stefani F. O. P. Moncalvo J.-M. Séguin A. Bérubé J. A. Hamelin R. C. (2009). Impact of an 8-year-old transgenic poplar plantation on the ectomycorrhizal fungal community. Appl. Environ. Microb. 75, 7527–7536. doi: 10.1128/aem.01120-09 PMC2786396 19801471

[B86] Sterkenburg E. Clemmensen K. E. Ekblad A. Finlay R. D. Lindahl B. D. (2018). Contrasting effects of ectomycorrhizal fungi on early and late stage decomposition in a boreal forest. Isme J. 12, 2187–2197. doi: 10.1038/s41396-018-0181-2 29880913 PMC6092328

[B87] Stone D. M. Elioff J. D. (1998). Soil properties and aspen development five years after compaction and forest floor removal. Can. J. Soil Sci. 78, 51–58. doi: 10.4141/s97-026

[B88] Tedersoo L. Bahram M. Zobel M. (2020). How mycorrhizal associations drive plant population and community biology. Science 367, eaba1223. doi: 10.1126/science.aba1223 32079744

[B89] Uroz S. Calvaruso C. Turpault M.-P. Frey-Klett P. (2009). Mineral weathering by bacteria: ecology, actors and mechanisms. Trends Microbiol. 17, 378–387. doi: 10.1016/j.tim.2009.05.004 19660952

[B90] Vadeboncoeur M. A. Hamburg S. P. Yanai R. D. Blum J. D. (2014). Rates of sustainable forest harvest depend on rotation length and weathering of soil minerals. For. Ecol. Manag. 318, 194–205. doi: 10.1016/j.foreco.2014.01.012

[B91] Van Der Heijden M. G. A. van der, Bardgett R. D. van Straalen N. M. (2008). The unseen majority: soil microbes as drivers of plant diversity and productivity in terrestrial ecosystems. Ecol. Lett. 11, 296–310. doi: 10.1111/j.1461-0248.2007.01139.x 18047587

[B92] van der Putten W. H. Bardgett R. D. Bever J. D. Bezemer T. M. Casper B. B. Fukami T.. (2013). Plant–soil feedbacks: the past, the present and future challenges. J. Ecol. 101, 265–276. doi: 10.1111/1365-2745.12054

[B93] van Schöll L. Kuyper T. W. Smits M. M. Landeweert R. Hoffland E. van Breemen N. (2008). Rock-eating mycorrhizas: their role in plant nutrition and biogeochemical cycles. Plant Soil 303, 35–47. doi: 10.1007/s11104-007-9513-0

[B94] Villarreal-Ruiz L. Neri-Luna C. (2017). Testing sampling effort and relative abundance descriptors of belowground ectomycorrhizal fungi in a UK planted scots pine woodland. Mycol 9, 1–10. doi: 10.1080/21501203.2017.1394393 PMC6059046 30123666

[B95] Wang Q. Garrity G. M. Tiedje J. M. Cole J. R. (2007). Naïve Bayesian classifier for rapid assignment of rRNA sequences into the new bacterial taxonomy. Appl. Environ. Microbiol. 73, 5261–5267. doi: 10.1128/AEM.00062-07 17586664 PMC1950982

[B96] Wei T. Simko V. (2017) R package “corrplot”: Visualization of a correlation matrix. Available at: https://github.com/taiyun/corrplot.

[B97] White T. J. Bruns T. Lee S. Taylor J. (1990). Amplification and direct sequencing of fungal ribosomal RNA genes for phylogenetics. In: Innis MA, Gelfand DH, Sninsky JJ, White TJ (eds) PCR protocols. (San Diego: Academic Press), 38, 315–322 doi: 10.1016/B978-0-12-372180-8.50042-1

[B98] Wickham H. (2007). Reshaping data with the reshape package. J. Stat. Softw. 21, 1–20.

[B99] Wickham H. (2011). The split-Apply-Combine strategy for data analysis. J. Stat. Softw. 40, 1–29. doi: 10.18637/jss.v040.i01

[B100] Wickham H. (2016). ggplot2: elegant graphics for data analysis. (Switzerland:Springer).

[B101] Wickham H. Girlich M. (2022) Tidyr: Tidy messy data. Available at: https://CRAN.R-project.org/package=tidyr.

[B102] Xie Y. (2014) Knitr: A comprehensive tool for reproducible research in r. Available at: http://www.crcpress.com/product/isbn/9781466561595.

[B103] Xie Y. (2015) Dynamic documents with r and knitr. Available at: https://yihui.org/knitr/.

[B104] Xie Y. (2022) Knitr: A general-purpose package for dynamic report generation in r. Available at: https://yihui.org/knitr/.

[B105] Xie Y. Allaire J. J. Grolemund G. (2018) R markdown: The definitive guide. Available at: https://bookdown.org/yihui/rmarkdown.

[B106] Xie Y. Dervieux C. Riederer E. (2020) R markdown cookbook. Available at: https://bookdown.org/yihui/rmarkdown-cookbook.

[B107] Zaharescu D. G. Burghelea C. I. Dontsova K. Reinhard C. T. Chorover J. Lybrand R. (2020). Biogeochemical cycles. Geophys. Monogr. Ser. 251, 3–32. doi: 10.1002/9781119413332.ch1

