# Supplementary material for: Does wood mulch trigger microbially mediated positive plant-soil feedback in degraded boreal forest sites? A post hoc study
Source: Front Plant Sci. 2023 May 3;14:1122445. doi: 10.3389/fpls.2023.1122445 (PMC10191178; doi:10.3389/fpls.2023.1122445)
Supplement: Supplementary file 1 [file DataSheet_1.docx]

Supplementary information

**Main features of the dataset.** A total of 4,080,470 and 7,717,937 high-quality 16S bacterial and ITS2 fungal sequences were obtained by sequencing 226–235 soil, rhizosphere and root samples (**Figure S4**). These sequences were clustered into 5,884 bacterial amplicon sequence variants (ASVs) and 561 fungal operational taxonomic units (OTUs). The number of bacterial ASVs and fungal OTUs reached saturation for each sampled compartment (i.e., for soil, rhizosphere and root samples, **Figures S5 and S6**). Prior to performing alpha-diversity calculations, the 16S and ITS2 datasets were rarefied to 10,000 and 15,000 sequences respectively, reducing the number of bacterial ASVs to 5,825 and the number of fungal OTUs to 559. The rarefaction step retained 53% and 42% of the bacterial and fungal sequences respectively and ~95% of the samples in each dataset (**Figure S7**).

**Table S1.** Evaluation of nutrient input from the Nutricote ® 18-6-8 fertilizer

|  | Nitrogen | Phosphorus | Potassium |
| --- | --- | --- | --- |
| Per tree (25 g) | 18 | 6 | 8 |
| g m^-2^ | 3.15 | 1.05 | 1.4 |
| kg ha^-1^ | 31.5 | 10.5 | 14 |

**Table S2.** Analysis of multivariate dispersion based on Bray-Curtis dissimilarities using abundance data (log[x+1] transformed) for bacteria and fungi (ASVs/OTUs and function abundance) in relation to the productivity levels (NPP, LPP and HPP) and sampled compartments (soil, rhizosphere, root). Since the null-productivity level only includes samples from the soil compartment, the interaction term was tested with low- and high-productivity levels only, and df = 2 (instead of df = 4).

|  | Bacteria ASVs | | | | Fungi OTUs | | | | Bacteria functions | | | | Fungi functions | | | |
| --- | --- | --- | --- | --- | --- | --- | --- | --- | --- | --- | --- | --- | --- | --- | --- | --- |
| Sources of variation | ndf | ddf | *F* | *p* | ndf | ddf | *F* | *p* | ndf | ddf | *F* | *p* | ndf | ddf | *F* | *p* |
| Productivity | 2 | 4.2 | 0.6 | 0.60 | 2 | 4.2 | 2.9 | 0.23 | 2 | 4.4 | 1.0 | 0.29 | 2 | 4.3 | 14.4 | **0.012** |
| Compartment | 2 | 9.9 | 11.7 | **0.002** | 2 | 10.1 | 5.4 | **0.033** | 2 | 10.1 | 0.8 | 0.47 | 2 | 10.0 | 0.1 | 0.88 |
| Productivity × compartment | 2 | 7.9 | 1.2 | 0.35 | 2 | 8.0 | 9.9 | **0.007** | 2 | 8.0 | 1.5 | 0.29 | 2 | 8.0 | 1.7 | 0.23 |
| Contrasts: |  |  |  |  |  |  | t-ratio | *p* |  |  |  |  |  |  | t-ratio | *p* |
| HPP Soil *vs* LPP Soil |  |  |  |  |  |  | -0.9 | 0.98 |  |  |  |  |  |  |  |  |
| HPP Root *vs* LPP Root |  |  |  |  |  |  | 3.6 | 0.14 |  |  |  |  |  |  |  |  |
| HPP Rhiz *vs* LPP Rhiz |  |  |  |  |  |  | 1.7 | 0.75 |  |  |  |  |  |  |  |  |
| HPP *vs* LPP |  |  |  |  |  |  | 1.8 | 0.37 |  |  |  |  |  |  | -5.1 | 0.07 |

ndf: numerator degrees of freedom; ddf: denominator degrees of freedom; *F*: Anova *F* statistic to compare (between different groups) the distances of observations to their group centroid; *p*: *p* value based on Monte Carlo random draw. Bold values indicate statistical significance at *p* < 0.05.

**Table S3.** List of bacterial ASVs with high differential rankings in samples from low-productivity plots (numerator, top 10% of ASVs, listed from highest to lowest) and in samples from high-productivity plots (denominator, bottom 10% of ASVs, listed from highest to lowest).

| ASV | Log-ratio | Phylum | Class | Order | Family | Genus | Species | Log-ratio classification |
| --- | --- | --- | --- | --- | --- | --- | --- | --- |
| ASV0098 | 6.01 | *Actinobacteria* | *Actinobacteria* | *Actinomycetales* | *Mycobacteriaceae* | *Mycobacterium* |  | Numerator |
| ASV0030 | 4.79 | *Acidobacteria* | *Acidobacteriia* | *Acidobacteriales* | *Koribacteraceae* | *Candidatus Koribacter* |  | Numerator |
| ASV0070 | 4.37 | *Proteobacteria* | *Gammaproteobacteria* | *Xanthomonadales* | *Sinobacteraceae* |  |  | Numerator |
| ASV0058 | 3.79 | *Bacteroidetes* | *[Saprospirae]* | *[Saprospirales]* | *Chitinophagaceae* |  |  | Numerator |
| ASV0036 | 3.70 | *Proteobacteria* | *Betaproteobacteria* | *Burkholderiales* | *Burkholderiaceae* | *Burkholderia* |  | Numerator |
| ASV0031 | 3.67 | *Acidobacteria* | *Acidobacteriia* | *Acidobacteriales* | *Acidobacteriaceae* |  |  | Numerator |
| ASV0042 | 2.91 | *Acidobacteria* | *Acidobacteriia* | *Acidobacteriales* | *Acidobacteriaceae* |  |  | Numerator |
| ASV0085 | 2.87 | *Bacteroidetes* | *Sphingobacteriia* | *Sphingobacteriales* | *Sphingobacteriaceae* |  |  | Numerator |
| ASV0046 | 2.76 | *Chloroflexi* | TK10 | B07_WMSP1 | FFCH4570 |  |  | Numerator |
| ASV0102 | 2.74 | *Bacteroidetes* | *Sphingobacteriia* | *Sphingobacteriales* | *Sphingobacteriaceae* |  |  | Numerator |
| ASV0048 | 2.59 | *Proteobacteria* | *Alphaproteobacteria* | *Rhizobiales* | *Methylocystaceae* |  |  | Numerator |
| ASV0089 | -7.79 | *Bacteroidetes* | *[Saprospirae]* | *[Saprospirales]* | *Chitinophagaceae* |  |  | Denominator |
| ASV0068 | -6.78 | *Actinobacteria* | *Actinobacteria* | *Actinomycetales* | *Streptomycetaceae* |  |  | Denominator |
| ASV0045 | -6.62 | *Proteobacteria* | *Gammaproteobacteria* | *Xanthomonadales* | *Sinobacteraceae* |  |  | Denominator |
| ASV0113 | -6.25 | *Actinobacteria* | *Actinobacteria* | *Actinomycetales* | *Streptomycetaceae* |  |  | Denominator |
| ASV0115 | -5.06 | *Proteobacteria* | *Alphaproteobacteria* | *Rhodospirillales* | *Acetobacteraceae* |  |  | Denominator |
| ASV0107 | -4.18 | *Bacteroidetes* | *[Saprospirae]* | *[Saprospirales]* | *Chitinophagaceae* |  |  | Denominator |
| ASV0092 | -3.44 | *Planctomycetes* | *Planctomycetia* | *Gemmatales* | *Isosphaeraceae* |  |  | Denominator |
| ASV0095 | -3.41 | *Bacteroidetes* | *[Saprospirae]* | *[Saprospirales]* | *Chitinophagaceae* |  |  | Denominator |
| ASV0120 | -3.19 | *Proteobacteria* | *Betaproteobacteria* | *Burkholderiales* | *Comamonadaceae* | *Rubrivivax* | *gelatinosus* | Denominator |
| ASV0038 | -3.18 | *Proteobacteria* | *Alphaproteobacteria* | *Rhizobiales* | *Bradyrhizobiaceae* |  |  | Denominator |
| ASV0078 | -2.73 | *Proteobacteria* | *Alphaproteobacteria* | *Rhodospirillales* | *Acetobacteraceae* |  |  | Denominator |

**Table S4.** List of fungal OTUs with high differential rankings in samples from the LPP (numerator, top 10% of OTUs, listed from highest to lowest log-ratio fold change values) and in samples from HPP (denominator, bottom 10% of OTUs, listed from highest to lowest).

| OTU | Log-ratio | Phylum | Class | Order | Family | Genus | Species | Log-Ratio classification |
| --- | --- | --- | --- | --- | --- | --- | --- | --- |
| OTU020 | 6.10 | *Ascomycota* | *Eurotiomycetes* | *Eurotiales* | *Trichocomaceae* | *Sagenomella* |  | Numerator |
| OTU046 | 5.65 | *Basidiomycota* | *Agaricomycetes* | *Thelephorales* | *Thelephoraceae* | *Tomentella* |  | Numerator |
| OTU049 | 5.32 | *Ascomycota* | *Eurotiomycetes* | *Chaetothyriales* | *Herpotrichiellaceae* |  |  | Numerator |
| OTU050 | 5.08 | *Basidiomycota* | *Agaricomycetes* | *Agaricales* | *Strophariaceae* | *Pholiota* | *Pholiota mixta* | Numerator |
| OTU041 | 4.96 | *Ascomycota* | *Leotiomycetes* |  |  |  |  | Numerator |
| OTU033 | 4.96 | *Basidiomycota* | *Agaricomycetes* | *Boletales* | *Melanogastraceae* | *Alpova* |  | Numerator |
| OTU059 | 4.61 | *Basidiomycota* | *Agaricomycetes* | *Russulales* | *Russulaceae* | *Lactarius* |  | Numerator |
| OTU027 | 4.41 | *Basidiomycota* | *Agaricomycetes* | *Boletales* | *Rhizopogonaceae* | *Rhizopogon* | *Rhizopogon pseudoroseolus* | Numerator |
| OTU025 | -6.12 | *Basidiomycota* | *Agaricomycetes* | *Agaricales* | *Cortinariaceae* | *Cortinarius* | *Cortinarius heterodepressus* | Denominator |
| OTU024 | -6.01 | *Ascomycota* | *Laboulbeniomycetes* | *Pyxidiophorales* |  |  |  | Denominator |
| OTU040 | -5.89 | *Basidiomycota* | *Agaricomycetes* | *Agaricales* | *Hymenogastraceae* | *Hebeloma* |  | Denominator |
| OTU013 | -5.56 | *Basidiomycota* | *Agaricomycetes* | *Atheliales* | *Atheliaceae* | *Piloderma* |  | Denominator |
| OTU044 | -5.26 | *Basidiomycota* | *Agaricomycetes* | *Agaricales* | *Cortinariaceae* | *Cortinarius* | *Cortinarius parvannulatus* | Denominator |
| OTU053 | -5.14 | *Basidiomycota* | *Agaricomycetes* | *Agaricales* | *Cortinariaceae* | *Cortinarius* | *Cortinarius comptulus* | Denominator |
| OTU087 | -4.61 | *Basidiomycota* | *Agaricomycetes* | *Thelephorales* | *Thelephoraceae* |  |  | Denominator |
| OTU034 | -4.47 | *Ascomycota* | *Saccharomycetes* | *Saccharomycetales* | *Trichomonascaceae* | *Sugiyamaella* | *Sugiyamaella paludigena* | Denominator |

**Table S5.** Log-ratio value of fungal OTUs from the genus *Cortinarius* (denominator) and the top 10% of OTUs (numerator), listed from highest to lowest log-ratio fold change values.

| OTU | Log-ratio | Phylum | Class | Order | Family | Genus | Species | Log-Ratio classification |
| --- | --- | --- | --- | --- | --- | --- | --- | --- |
| OTU020 | 6.10 | *Ascomycota* | *Eurotiomycetes* | *Eurotiales* | *Trichocomaceae* | *Sagenomella* |  | Numerator |
| OTU046 | 5.65 | *Basidiomycota* | *Agaricomycetes* | *Thelephorales* | *Thelephoraceae* | *Tomentella* | *Tomentella ellisii* | Numerator |
| OTU049 | 5.32 | *Ascomycota* | *Eurotiomycetes* | *Chaetothyriales* | *Herpotrichiellaceae* | unidentified |  | Numerator |
| OTU050 | 5.08 | *Basidiomycota* | *Agaricomycetes* | *Agaricales* | *Strophariaceae* | *Pholiota* | *Pholiota mixta* | Numerator |
| OTU041 | 4.96 | *Ascomycota* | *Leotiomycetes* |  |  |  |  | Numerator |
| OTU033 | 4.96 | *Basidiomycota* | *Agaricomycetes* | *Boletales* | *Melanogastraceae* | *Alpova* |  | Numerator |
| OTU059 | 4.61 | *Basidiomycota* | *Agaricomycetes* | *Russulales* | *Russulaceae* | *Lactarius* |  | Numerator |
| OTU027 | 4.41 | *Basidiomycota* | *Agaricomycetes* | *Boletales* | *Rhizopogonaceae* | *Rhizopogon* | *Rhizopogon pseudoroseolus* | Numerator |
| OTU025 | -6.12 | *Basidiomycota* | *Agaricomycetes* | *Agaricales* | *Cortinariaceae* | *Cortinarius* | *Cortinarius heterodepressus* | Denominator |
| OTU044 | -5.26 | *Basidiomycota* | *Agaricomycetes* | *Agaricales* | *Cortinariaceae* | *Cortinarius* | *Cortinarius parvannulatus* | Denominator |
| OTU053 | -5.14 | *Basidiomycota* | *Agaricomycetes* | *Agaricales* | *Cortinariaceae* | *Cortinarius* | *Cortinarius comptulus* | Denominator |
| OTU055 | -3.94 | *Basidiomycota* | *Agaricomycetes* | *Agaricales* | *Cortinariaceae* | *Cortinarius* | *Cortinarius athabascus* | Denominator |
| OTU018 | -3.30 | *Basidiomycota* | *Agaricomycetes* | *Agaricales* | *Cortinariaceae* | *Cortinarius* | *Cortinarius biformis* | Denominator |
| OTU035 | -2.74 | *Basidiomycota* | *Agaricomycetes* | *Agaricales* | *Cortinariaceae* | *Cortinarius* | *Cortinarius scandens* | Denominator |
| OTU015 | -2.59 | *Basidiomycota* | *Agaricomycetes* | *Agaricales* | *Cortinariaceae* | *Cortinarius* | *Cortinarius croceus* | Denominator |
| OTU070 | -1.74 | *Basidiomycota* | *Agaricomycetes* | *Agaricales* | *Cortinariaceae* | *Cortinarius* |  | Denominator |
| OTU066 | -0.80 | *Basidiomycota* | *Agaricomycetes* | *Agaricales* | *Cortinariaceae* | *Cortinarius* |  | Denominator |
| OTU021 | 1.39 | *Basidiomycota* | *Agaricomycetes* | *Agaricales* | *Cortinariaceae* | *Cortinarius* |  | Numerator |

**Table S6.** List of nodes from high-productivity plots (HPP) and low-productivity plots (LPP) networks (Figure 7) with > 1 interaction and their taxonomic assignment. “F” and “B” refer to fungal OTUs and bacterial ASVs, respectively.

| Taxa | Interaction | Productivity | Kingdom | Phylum | Class | Order | Family | Genus | Species |
| --- | --- | --- | --- | --- | --- | --- | --- | --- | --- |
| F69 | 14 | HPP | *Fungi* | *Ascomycota* | *Saccharomycetes* | *Saccharomycetales* |  |  |  |
| F5 | 12 | HPP | *Fungi* | *Ascomycota* |  |  |  |  |  |
| F47 | 12 | HPP | *Fungi* | *Mortierellomycota* | *Mortierellomycetes* | *Mortierellales* | *Mortierellaceae* | *Mortierella* |  |
| F65 | 12 | HPP | *Fungi* | *Mucoromycota* | *Umbelopsidomycetes* | *Umbelopsidales* | *Umbelopsidaceae* | *Umbelopsis* |  |
| F10 | 11 | HPP | *Fungi* | *Mucoromycota* | *Umbelopsidomycetes* | *Umbelopsidales* | *Umbelopsidaceae* | *Umbelopsis* |  |
| B87 | 11 | HPP | *Bacteria* | *Proteobacteria* | *Alphaproteobacteria* | Ellin 329 |  |  |  |
| F29 | 10 | HPP | *Fungi* | *Mortierellomycota* | *Mortierellomycetes* | *Mortierellales* | *Mortierellaceae* | *Mortierella* | *M. basiparvispora* |
| F81 | 10 | HPP | *Fungi* | *Basidiomycota* | *Tremellomycetes* | *Filobasidiales* | *Piskurozymaceae* | *Solicoccozyma* | *S. terricola* |
| B101 | 10 | HPP | *Bacteria* | *Proteobacteria* | *Alphaproteobacteria* | *Rhodospirillales* | *Acetobacteraceae* | *Acidisoma* |  |
| F31 | 9 | HPP | *Fungi* | *Ascomycota* | *Eurotiomycetes* | *Eurotiales* | *Aspergillaceae* | *Penicillium* |  |
| F38 | 8 | HPP | *Fungi* | *Ascomycota* | *Eurotiomycetes* | *Chaetothyriales* | *Herpotrichiellaceae* | *Cladophialophora* | *C. minutissima* |
| B7 | 8 | HPP | *Bacteria* | *Proteobacteria* | *Gammaproteobacteria* | *Xanthomonadales* | *Sinobacteraceae* |  |  |
| F28 | 7 | HPP | *Fungi* | *Basidiomycota* | *Microbotryomycetes* | *Leucosporidiales* |  |  |  |
| B23 | 7 | HPP | *Bacteria* | *Proteobacteria* | *Alphaproteobacteria* | *Rhizobiales* | *Bradyrhizobiaceae* | *Bradyrhizobium* |  |
| F1 | 6 | HPP | *Fungi* | *Basidiomycota* | *Agaricomycetes* | *Cantharellales* | *Incertae sedis* | *Sistotrema* |  |
| F45 | 6 | HPP | *Fungi* | *Basidiomycota* | *Tremellomycetes* | *Filobasidiales* | *Piskurozymaceae* | *Solicoccozyma* |  |
| B27 | 6 | HPP | *Bacteria* | *Acidobacteria* | *Acidobacteriia* | *Acidobacteriales* | *Acidobacteriaceae* | *Granulicella* | *G. mallensis* |
| B50 | 6 | HPP | *Bacteria* | *Bacteroidetes* | *Sphingobacteriia* | *Sphingobacteriales* | *Sphingobacteriaceae* |  |  |
| B56 | 6 | HPP | *Bacteria* | *Actinobacteria* | *Actinobacteria* | *Actinomycetales* | *Microbacteriaceae* | *Salinibacterium* |  |
| B125 | 6 | HPP | *Bacteria* | *Proteobacteria* | *Alphaproteobacteria* | Ellin 329 |  |  |  |
| B13 | 4 | HPP | *Bacteria* | *Proteobacteria* | *Gammaproteobacteria* | *Xanthomonadales* | *Sinobacteraceae* |  |  |
| B22 | 4 | HPP | *Bacteria* | *Proteobacteria* | *Betaproteobacteria* | *Burkholderiales* | *Burkholderiaceae* | *Burkholderia* |  |
| F11 | 3 | HPP | *Fungi* | *Ascomycota* | *Leotiomycetes* | *Helotiales* | *Myxotrichaceae* | *Oidiodendron* |  |
| B1 | 3 | HPP | *Bacteria* | *Proteobacteria* | *Betaproteobacteria* | *Burkholderiales* | *Burkholderiaceae* | *Burkholderia* | *B. glathei* |
| B11 | 3 | HPP | *Bacteria* | *Proteobacteria* | *Alphaproteobacteria* | *Rhizobiales* | *Hyphomicrobiaceae* | *Rhodoplanes* |  |
| B16 | 3 | HPP | *Bacteria* | *Proteobacteria* | *Alphaproteobacteria* | *Rhizobiales* | *Rhizobiaceae* | *Rhizobium* |  |
| B20 | 3 | HPP | *Bacteria* | *Proteobacteria* | *Betaproteobacteria* | *Burkholderiales* | *Burkholderiaceae* | *Burkholderia* | *B. bryophila* |
| B24 | 3 | HPP | *Bacteria* | *Actinobacteria* | *Actinobacteria* | *Actinomycetales* | *Mycobacteriaceae* | *Mycobacterium* | *M. celatum* |
| B3 | 2 | HPP | *Bacteria* | *Bacteroidetes* | *[Saprospirae]* | *[Saprospirales]* | *Chitinophagaceae* |  |  |
| B51 | 2 | HPP | *Bacteria* | *Proteobacteria* | *Betaproteobacteria* | *Burkholderiales* | *Burkholderiaceae* | *Burkholderia* | *B. bryophila* |
| B78 | 2 | HPP | *Bacteria* | *Proteobacteria* | *Alphaproteobacteria* | *Rhodospirillales* | *Acetobacteraceae* |  |  |
| B109 | 2 | HPP | *Bacteria* | *Acidobacteria* | *Solibacteres* | *Solibacterales* |  |  |  |
| B133 | 2 | HPP | *Bacteria* | *Proteobacteria* | *Alphaproteobacteria* | *Rhizobiales* | *Methylocystaceae* |  |  |
| B191 | 2 | HPP | *Bacteria* | *Actinobacteria* | *Actinobacteria* | *Actinomycetales* | *Mycobacteriaceae* | *Mycobacterium* |  |
| F7 | 1 | HPP | *Fungi* | *Basidiomycota* | *Agaricomycetes* | *Boletales* | *Suillaceae* | *Suillus* | *S. tomentosus* |
| B4 | 1 | HPP | *Bacteria* | *Proteobacteria* | *Gammaproteobacteria* | *Xanthomonadales* | *Sinobacteraceae* |  |  |
| B9 | 1 | HPP | *Bacteria* | *Bacteroidetes* | *[Saprospirae]* | *[Saprospirales]* | *Chitinophagaceae* |  |  |
| B18 | 1 | HPP | *Bacteria* | *Proteobacteria* | *Gammaproteobacteria* | *Xanthomonadales* | *Sinobacteraceae* |  |  |
| B37 | 1 | HPP | *Bacteria* | *Actinobacteria* | *Actinobacteria* | *Actinomycetales* | *Mycobacteriaceae* | *Mycobacterium* |  |
| F20 | 5 | LPP | *Fungi* | *Ascomycota* | *Eurotiomycetes* | *Eurotiales* | *Trichocomaceae* | *Sagenomella* |  |
| F6 | 4 | LPP | *Fungi* | *Basidiomycota* | *Agaricomycetes* | *Boletales* | *Suillaceae* | *Suillus* | *S. glandulosipes* |
| F8 | 4 | LPP | *Fungi* | *Basidiomycota* | *Agaricomycetes* | *Cantharellales* | *Incertae sedis* | *Sistotrema* |  |
| F3 | 3 | LPP | *Fungi* | *Ascomycota* | *Leotiomycetes* | *Helotiales* | *Vibrisseaceae* | *Phialocephala* |  |
| F1 | 2 | LPP | *Fungi* | *Basidiomycota* | *Agaricomycetes* | *Cantharellales* | *Incertae sedis* | *Sistotrema* |  |
| F38 | 2 | LPP | *Fungi* | *Ascomycota* | *Eurotiomycetes* | *Chaetothyriales* | *Herpotrichiellaceae* | *Cladophialophora* | *C. minutissima* |
| B8 | 2 | LPP | *Bacteria* | *Proteobacteria* | *Gammaproteobacteria* | *Xanthomonadales* | *Sinobacteraceae* |  |  |
| B16 | 1 | LPP | *Bacteria* | *Proteobacteria* | *Alphaproteobacteria* | *Rhizobiales* | *Rhizobiaceae* | *Rhizobium* |  |
| B42 | 1 | LPP | *Bacteria* | *Acidobacteria* | *Acidobacteriia* | *Acidobacteriales* | *Acidobacteriaceae* |  |  |

**Table S7.** Taxonomic assignment of the 87 core bacterial ASVs.

| **ASV** | **Phylum** | **Class** | **Order** | **Family** | **Genus** | **Species** |
| --- | --- | --- | --- | --- | --- | --- |
| ASV0001 | *Proteobacteria* | *Betaproteobacteria* | *Burkholderiales* | *Burkholderiaceae* | *Burkholderia* | *glathei* |
| ASV0002 | *Cyanobacteria* | *Chloroplast* | *Streptophyta* |  |  |  |
| ASV0003 | *Proteobacteria* | *Alphaproteobacteria* | *Rhizobiales* | *Bradyrhizobiaceae* |  |  |
| ASV0004 | *Bacteroidetes* | *[Saprospirae]* | *[Saprospirales]* | *Chitinophagaceae* |  |  |
| ASV0005 | *Proteobacteria* | *Gammaproteobacteria* | *Xanthomonadales* | *Sinobacteraceae* |  |  |
| ASV0007 | *Proteobacteria* | *Gammaproteobacteria* | *Xanthomonadales* | *Sinobacteraceae* |  |  |
| ASV0008 | *Proteobacteria* | *Gammaproteobacteria* | *Xanthomonadales* | *Sinobacteraceae* |  |  |
| ASV0009 | *Bacteroidetes* | *[Saprospirae]* | *[Saprospirales]* | *Chitinophagaceae* |  |  |
| ASV0010 | *Bacteroidetes* | *[Saprospirae]* | *[Saprospirales]* | *Chitinophagaceae* |  |  |
| ASV0011 | *Proteobacteria* | *Betaproteobacteria* | *Burkholderiales* | *Burkholderiaceae* | *Burkholderia* | *glathei* |
| ASV0013 | *Bacteroidetes* | *[Saprospirae]* | *[Saprospirales]* | *Chitinophagaceae* |  |  |
| ASV0014 | *Acidobacteria* | *Acidobacteriia* | *Acidobacteriales* | *Acidobacteriaceae* |  |  |
| ASV0016 | *Proteobacteria* | *Alphaproteobacteria* | *Rhizobiales* | *Bradyrhizobiaceae* | *Bradyrhizobium* |  |
| ASV0018 | *Proteobacteria* | *Gammaproteobacteria* | *Xanthomonadales* | *Sinobacteraceae* |  |  |
| ASV0019 | *Proteobacteria* | *Betaproteobacteria* | *Burkholderiales* | *Burkholderiaceae* | *Burkholderia* | *bryophila* |
| ASV0020 | *Acidobacteria* | *Acidobacteriia* | *Acidobacteriales* | *Acidobacteriaceae* | *Edaphobacter* |  |
| ASV0022 | *Proteobacteria* | *Gammaproteobacteria* | *Xanthomonadales* | *Sinobacteraceae* |  |  |
| ASV0023 | *Bacteroidetes* | *[Saprospirae]* | *[Saprospirales]* | *Chitinophagaceae* | *Flavisolibacter* |  |
| ASV0024 | *Acidobacteria* | *DA052* | *Ellin6513* |  |  |  |
| ASV0027 | *Actinobacteria* | *Actinobacteria* | *Actinomycetales* | *Mycobacteriaceae* | *Mycobacterium* | *celatum* |
| ASV0037 | *Acidobacteria* | *Acidobacteriia* | *Acidobacteriales* | *Acidobacteriaceae* |  |  |
| ASV0039 | *Proteobacteria* | *Alphaproteobacteria* | *Rhizobiales* | *Bradyrhizobiaceae* |  |  |
| ASV0040 | *Actinobacteria* | *Actinobacteria* | *Actinomycetales* | *Actinospicaceae* |  |  |
| ASV0050 | *Proteobacteria* | *Betaproteobacteria* | *Burkholderiales* | *Burkholderiaceae* | *Burkholderia* | *bryophila* |
| ASV0051 | *Bacteroidetes* | *Sphingobacteriia* | *Sphingobacteriales* | *Sphingobacteriaceae* |  |  |
| ASV0055 | *Proteobacteria* | *Alphaproteobacteria* | *Rhizobiales* | *Hyphomicrobiaceae* | *Rhodoplanes* |  |
| ASV0056 | *Proteobacteria* | *Gammaproteobacteria* | *Xanthomonadales* | *Sinobacteraceae* |  |  |
| ASV0078 | *Bacteroidetes* | *[Saprospirae]* | *[Saprospirales]* | *Chitinophagaceae* |  |  |
| ASV0087 | *Acidobacteria* | *Acidobacteriia* | *Acidobacteriales* | *Acidobacteriaceae* | *Edaphobacter* |  |
| ASV0091 | *Proteobacteria* | *Gammaproteobacteria* | *Xanthomonadales* | *Sinobacteraceae* |  |  |
| ASV0094 | *Proteobacteria* | *Alphaproteobacteria* | *Rhodospirillales* | *Acetobacteraceae* |  |  |
| ASV0101 | *Acidobacteria* | *DA052* | *Ellin6513* |  |  |  |
| ASV0109 | *Proteobacteria* | *Alphaproteobacteria* | *Rhodospirillales* | *Acetobacteraceae* |  |  |
| ASV0125 | *Proteobacteria* | *Betaproteobacteria* | *Burkholderiales* | *Oxalobacteraceae* |  |  |
| ASV0133 | *Acidobacteria* | *DA052* | *Ellin6513* |  |  |  |
| ASV0191 | *Cyanobacteria* | *Nostocophycideae* | *Nostocales* | *Nostocaceae* | *Stigonema* | *ocellatum* |
| ASV0012 | *Proteobacteria* | *Alphaproteobacteria* | *Rhizobiales* | *Hyphomicrobiaceae* | *Rhodoplanes* |  |
| ASV0017 | *Proteobacteria* | *Alphaproteobacteria* | *Rhizobiales* | *Rhizobiaceae* | *Rhizobium* |  |
| ASV0021 | *Actinobacteria* | *Actinobacteria* | *Actinomycetales* | *Mycobacteriaceae* | *Mycobacterium* |  |
| ASV0025 | *Proteobacteria* | *Betaproteobacteria* | *Burkholderiales* | *Burkholderiaceae* | *Burkholderia* |  |
| ASV0028 | *Cyanobacteria* | *Chloroplast* | *Streptophyta* |  |  |  |
| ASV0029 | *Chloroflexi* | *Ktedonobacteria* | *Ktedonobacterales* | *Ktedonobacteraceae* |  |  |
| ASV0033 | *Cyanobacteria* | *Chloroplast* | *Streptophyta* |  |  |  |
| ASV0042 | *Bacteroidetes* | *Sphingobacteriia* | *Sphingobacteriales* | *Sphingobacteriaceae* |  |  |
| ASV0046 | *Actinobacteria* | *Actinobacteria* | *Actinomycetales* | *Actinospicaceae* |  |  |
| ASV0048 | *Proteobacteria* | *Alphaproteobacteria* | *Rhodospirillales* | *Acetobacteraceae* |  |  |
| ASV0049 | *Acidobacteria* | *Acidobacteriia* | *Acidobacteriales* | *Acidobacteriaceae* |  |  |
| ASV0064 | *Proteobacteria* | *Betaproteobacteria* | *Burkholderiales* | *Oxalobacteraceae* | *Massilia* |  |
| ASV0082 | *Proteobacteria* | *Alphaproteobacteria* | *Rhodospirillales* | *Acetobacteraceae* |  |  |
| ASV0104 | *Bacteroidetes* | *Sphingobacteriia* | *Sphingobacteriales* | *Sphingobacteriaceae* |  |  |
| ASV0128 | *Acidobacteria* | *Acidobacteriia* | *Acidobacteriales* | *Koribacteraceae* |  |  |
| ASV0140 | *Proteobacteria* | *Deltaproteobacteria* | *Myxococcales* |  |  |  |
| ASV0030 | *Acidobacteria* | *Acidobacteriia* | *Acidobacteriales* | *Acidobacteriaceae* | *Granulicella* | *mallensis* |
| ASV0041 | *Proteobacteria* | *Alphaproteobacteria* | *Rhodospirillales* | *Rhodospirillaceae* | *Reyranella* | *massiliensis* |
| ASV0066 | *Acidobacteria* | *Acidobacteriia* | *Acidobacteriales* | *Acidobacteriaceae* |  |  |
| ASV0083 | *Actinobacteria* | *Actinobacteria* | *Actinomycetales* | *Streptomycetaceae* | *Streptacidiphilus* |  |
| ASV0090 | *Proteobacteria* | *Alphaproteobacteria* | *Rhizobiales* | *Bradyrhizobiaceae* | *Bosea* | *genosp.* |
| ASV0099 | *Proteobacteria* | *Gammaproteobacteria* | *Xanthomonadales* | *Sinobacteraceae* |  |  |
| ASV0114 | *Proteobacteria* | *Alphaproteobacteria* | *Rhodospirillales* | *Rhodospirillaceae* |  |  |
| ASV0116 | *Bacteroidetes* | *[Saprospirae]* | *[Saprospirales]* | *Chitinophagaceae* |  |  |
| ASV0124 | *Acidobacteria* | *Acidobacteriia* | *Acidobacteriales* | *Acidobacteriaceae* |  |  |
| ASV0132 | *Actinobacteria* | *Actinobacteria* | *Actinomycetales* | *Mycobacteriaceae* | *Mycobacterium* | *celatum* |
| ASV0134 | *Actinobacteria* | *Actinobacteria* | *Actinomycetales* | *Streptomycetaceae* |  |  |
| ASV0136 | *Proteobacteria* | *Alphaproteobacteria* | *Rhodospirillales* | *Acetobacteraceae* |  |  |
| ASV0139 | *Proteobacteria* | *Alphaproteobacteria* | *Caulobacterales* | *Caulobacteraceae* | *Phenylobacterium* |  |
| ASV0143 | *Planctomycetes* | *Phycisphaerae* | *WD2101* |  |  |  |
| ASV0188 | *Actinobacteria* | *Thermoleophilia* | *Solirubrobacterales* | *Conexibacteraceae* |  |  |
| ASV0194 | *Acidobacteria* | *DA052* | *Ellin6513* |  |  |  |
| ASV0205 | *Proteobacteria* | *Alphaproteobacteria* | *Rhizobiales* | *Hyphomicrobiaceae* | *Rhodoplanes* |  |
| ASV0214 | *Proteobacteria* | *Alphaproteobacteria* | *Rhodospirillales* | *Rhodospirillaceae* | *Reyranella* | *massiliensis* |
| ASV0215 | *Proteobacteria* | *Betaproteobacteria* | *Burkholderiales* | *Oxalobacteraceae* | *Janthinobacterium* |  |
| ASV0220 | *Proteobacteria* | *Alphaproteobacteria* | *Rhizobiales* | *Phyllobacteriaceae* | *Aminobacter* |  |
| ASV0226 | *Planctomycetes* | *Planctomycetia* | *Gemmatales* | *Isosphaeraceae* |  |  |
| ASV0238 | *Bacteroidetes* | *Sphingobacteriia* | *Sphingobacteriales* | *Sphingobacteriaceae* |  |  |
| ASV0251 | *Proteobacteria* | *Betaproteobacteria* | *Burkholderiales* | *Oxalobacteraceae* | *Herminiimonas* |  |
| ASV0258 | *Planctomycetes* | *Planctomycetia* | *Gemmatales* | *Gemmataceae* |  |  |
| ASV0269 | *Acidobacteria* | *DA052* | *Ellin6513* |  |  |  |
| ASV0281 | *Bacteroidetes* | *Sphingobacteriia* | *Sphingobacteriales* | *Sphingobacteriaceae* |  |  |
| ASV0304 | *Chloroflexi* | *Ktedonobacteria* | *Thermogemmatisporales* | *Thermogemmatisporaceae* |  |  |
| ASV0312 | *Firmicutes* | *Bacilli* | *Bacillales* | *Paenibacillaceae* | *Paenibacillus* |  |
| ASV0316 | *Proteobacteria* | *Alphaproteobacteria* | *Caulobacterales* | *Caulobacteraceae* | *Phenylobacterium* |  |
| ASV0442 | *Proteobacteria* | *Gammaproteobacteria* | *Xanthomonadales* | *Sinobacteraceae* |  |  |
| ASV0445 | *Planctomycetes* | *Planctomycetia* | *Gemmatales* | *Gemmataceae* |  |  |
| ASV0466 | *Acidobacteria* | *DA052* | *Ellin6513* |  |  |  |
| ASV0500 | *Bacteroidetes* | *[Saprospirae]* | *[Saprospirales]* | *Chitinophagaceae* | *Sediminibacterium* |  |
| ASV0555 | *Proteobacteria* | *Betaproteobacteria* | *MND1* |  |  |  |
| ASV0645 | *Proteobacteria* | *Alphaproteobacteria* | *Rhodospirillales* | *Acetobacteraceae* |  |  |

**Table S8.** Taxonomic assignment of the 24 core fungal OTUs.

| **OTU** | **Phylum** | **Class** | **Order** | **Family** | **Genus** | **Species** |
| --- | --- | --- | --- | --- | --- | --- |
| OTU001 | *Basidiomycota* | *Agaricomycetes* | *Cantharellales* | *Incertae sedis* | *Sistotrema* |  |
| OTU003 | *Ascomycota* | *Leotiomycetes* | *Helotiales* |  |  |  |
| OTU005 | *Ascomycota* |  |  |  |  |  |
| OTU007 | *Basidiomycota* | *Agaricomycetes* | *Boletales* | *Suillaceae* | *Suillus* | *tomentosus* |
| OTU010 | *Mucoromycota* | *Umbelopsidomycetes* | *Umbelopsidales* | *Umbelopsidaceae* | *Umbelopsis* |  |
| OTU011 | *Ascomycota* | *Leotiomycetes* | *Helotiales* | *Myxotrichaceae* | *Oidiodendron* |  |
| OTU028 | *Basidiomycota* | *Tremellomycetes* | *Trichosporonales* | *Trichosporonaceae* | *Apiotrichum* |  |
| OTU029 | *Basidiomycota* | *Agaricomycetes* | *Boletales* | *Suillaceae* | *Suillus* | *grevillei* |
| OTU031 | *Ascomycota* | *Eurotiomycetes* | *Eurotiales* | *Aspergillaceae* | *Penicillium* |  |
| OTU038 | *Ascomycota* | *Leotiomycetes* | *Helotiales* | *Dermateaceae* | *Cryptosporiopsis* |  |
| OTU045 | *Basidiomycota* | *Tritirachiomycetes* | *Tritirachiales* | *Tritirachiaceae* | *Paratritirachium* |  |
| OTU047 | *Ascomycota* | *Eurotiomycetes* | *Eurotiales* | *Trichocomaceae* | *Sagenomella* |  |
| OTU065 | *Ascomycota* | *Pezizomycetes* | *Pezizales* | *Pyronemataceae* | *Wilcoxina* | *mikolae* |
| OTU069 | *Ascomycota* | *Eurotiomycetes* | *Chaetothyriales* | *Herpotrichiellaceae* | *Cladophialophora* | *chaetospira* |
| OTU072 | *Mucoromycota* | *Umbelopsidomycetes* | *Umbelopsidales* | *Umbelopsidaceae* | *Umbelopsis* |  |
| OTU081 | *Basidiomycota* | *Tremellomycetes* | *Filobasidiales* | *Piskurozymaceae* | *Solicoccozyma* | *terricola* |
| OTU006 | *Basidiomycota* | *Agaricomycetes* | *Boletales* | *Suillaceae* | *Suillus* | *glandulosipes* |
| OTU008 | *Basidiomycota* | *Agaricomycetes* | *Cantharellales* | *Incertae sedis* | *Sistotrema* |  |
| OTU020 | *Ascomycota* | *Laboulbeniomycetes* | *Pyxidiophorales* |  |  |  |
| OTU002 | *Ascomycota* | *Leotiomycetes* | *Helotiales* | *Vibrisseaceae* | *Phialocephala* |  |
| OTU004 | *Basidiomycota* | *Agaricomycetes* | *Boletales* | *Suillaceae* | *Suillus* | *brevipes* |
| OTU052 | *Ascomycota* | *Eurotiomycetes* | *Chaetothyriales* | *Herpotrichiellaceae* |  |  |
| OTU102 | *Basidiomycota* | *Agaricomycetes* | *Cantharellales* | *Incertae sedis* | *Sistotrema* |  |
| OTU123 | *Mucoromycota* |  |  |  |  |  |

**
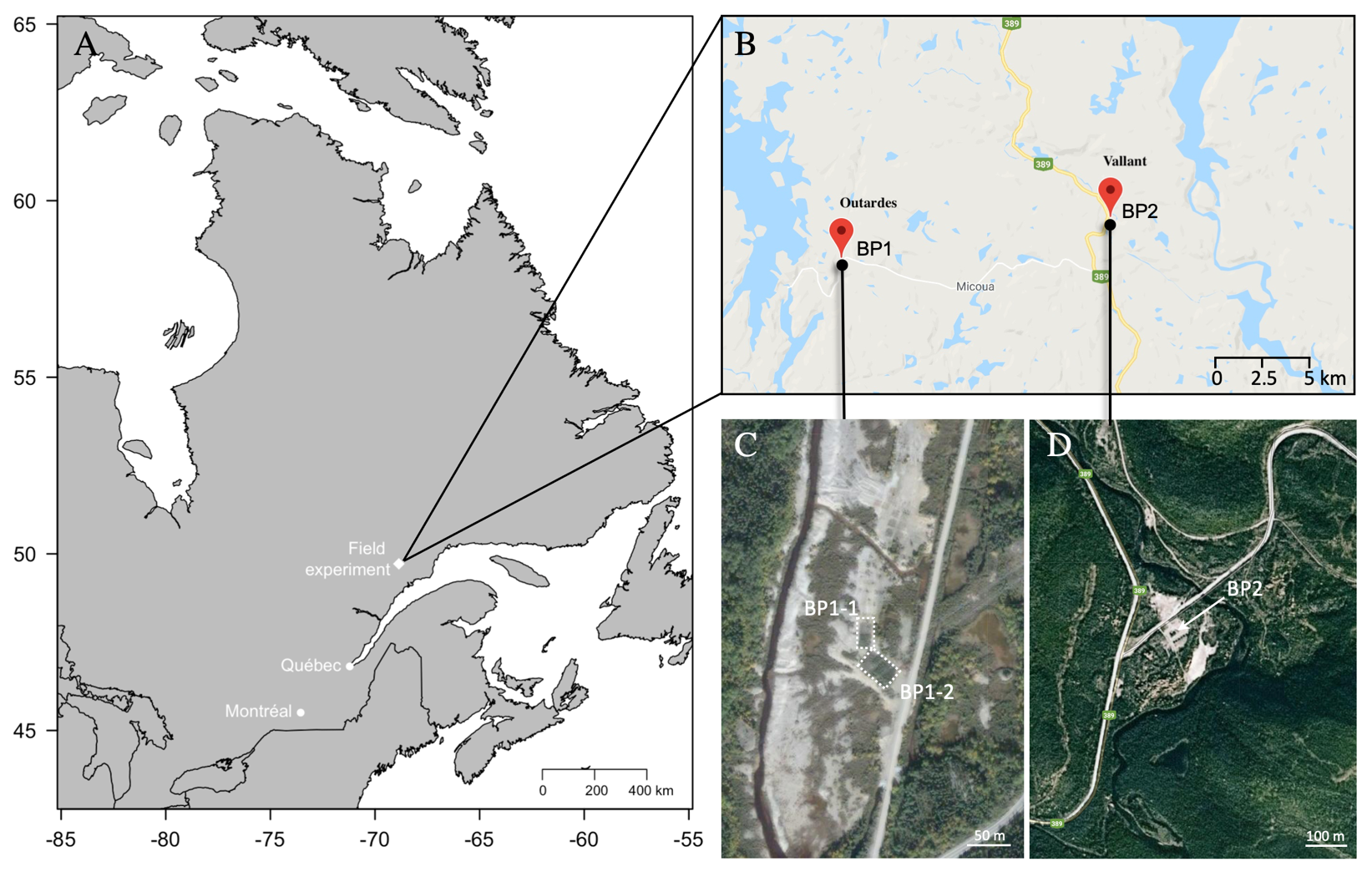
Figure S1.** (A) The field experiment is located 366 km northeast of Quebec City in the boreal forest. (B) BP1 and BP2 are 13 km apart. (C) and (D) show an aerial view of the three borrow pits, BP1-1 and BP-1-2 (C), and BP2 (D).

**
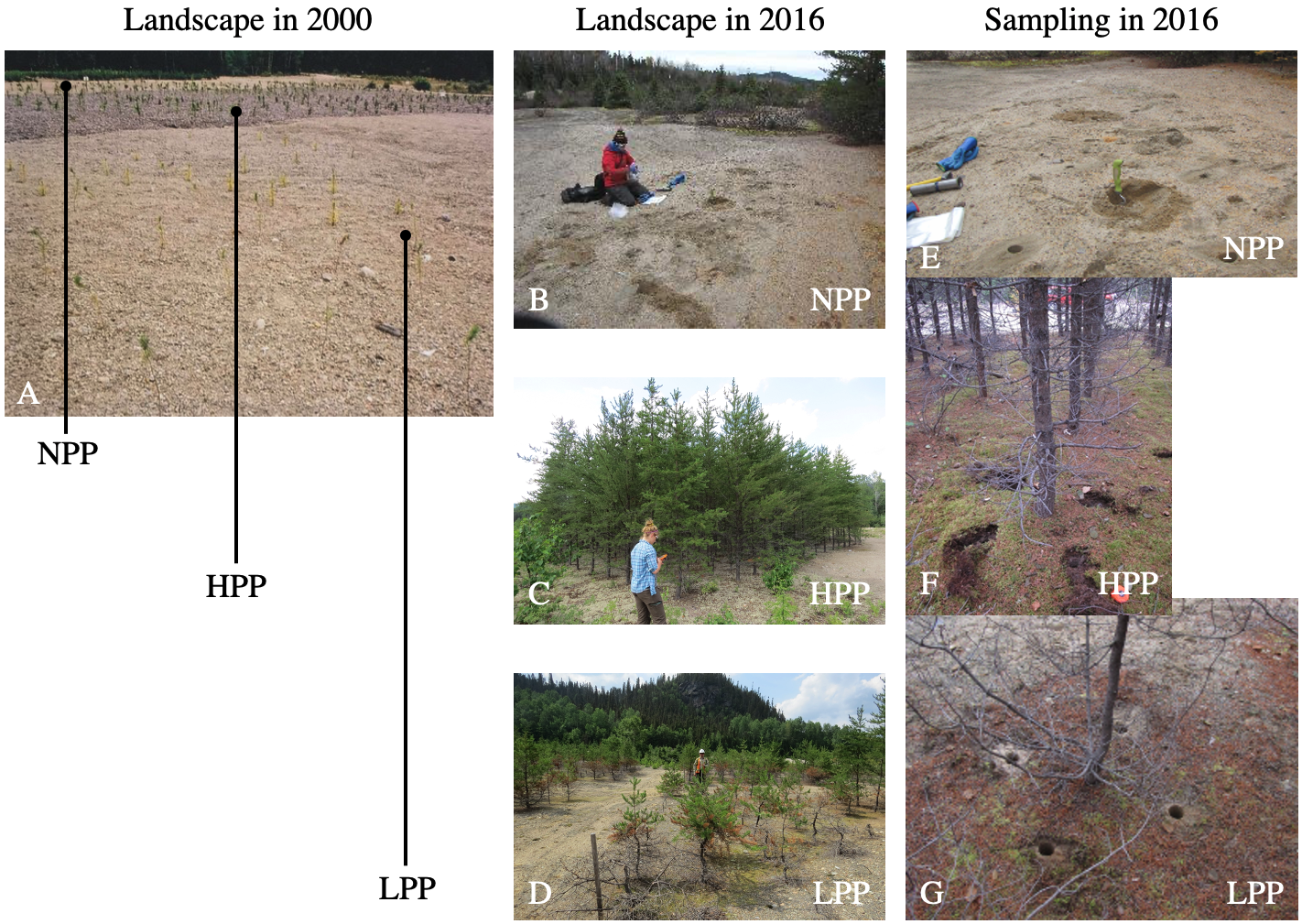
Figure S2.** (A) General view of the plots in 2000. From back to front, the picture shows the three experimental units: an unamended plot with no surviving tree (null-productivity plots – NPP), a plot amended with a continuous cover (2–3 cm in thickness) of locally produced RCW (high-productivity plots – HPP), a plot where ~3 L of RCW was applied only at the seedling base after planting (low-productivity plots – LPP). Mulch around the seedlings in the LPP was not applied at the time of the photo. (B), (C) and (D): General view of plots in 2016 for each productivity level. (E) to (G) Sampling in each plot type.

**
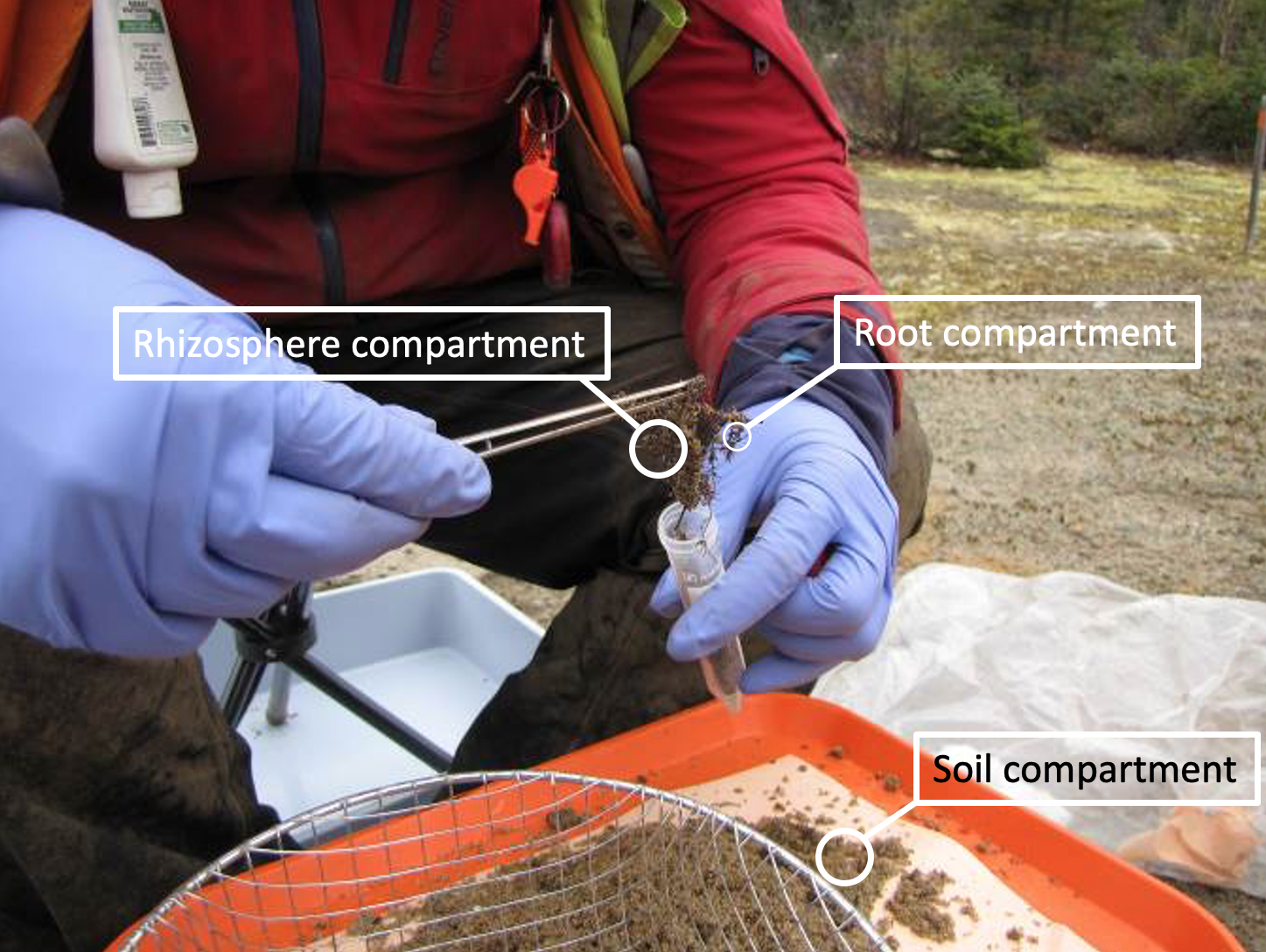
Figure S3.** Sampling the belowground microbiome: sieved bulk soil was used to target the soil compartment. Root-attached soil was used to target the rhizosphere compartment. Clean root tissue was used to target the root compartment.


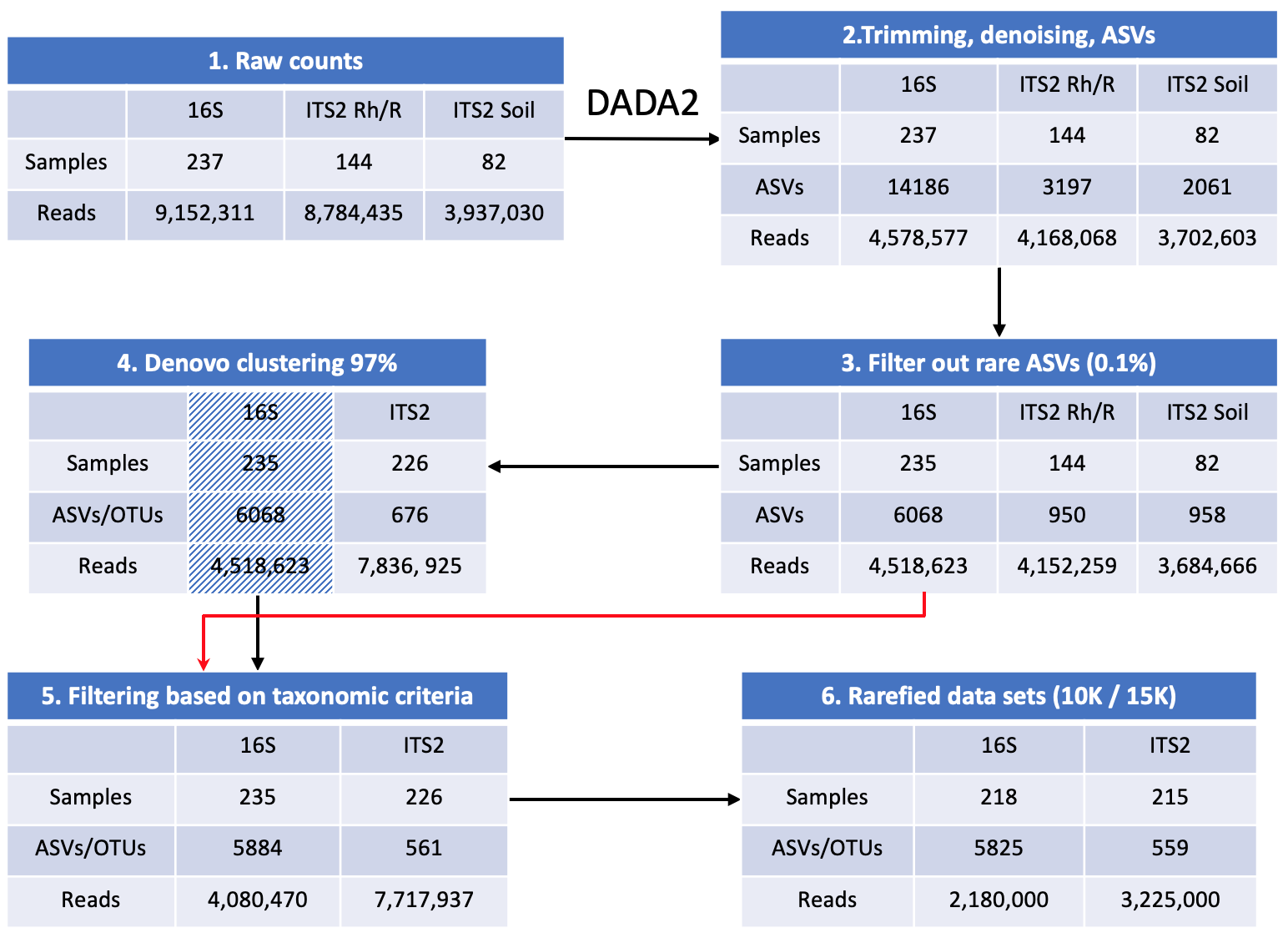
Figure S4. Representation of how the number of samples, sequences, bacterial ASVs and fungal OTUs evolved during the bioinformatics process, from raw data to feature tables used for diversity analyses. The red arrow indicates that *de novo* clustering was skipped to generate the final 16S feature table. The bacterial and fungal datasets were rarefied using a threshold of 10,000 and 15,000 sequences per sample, respectively.


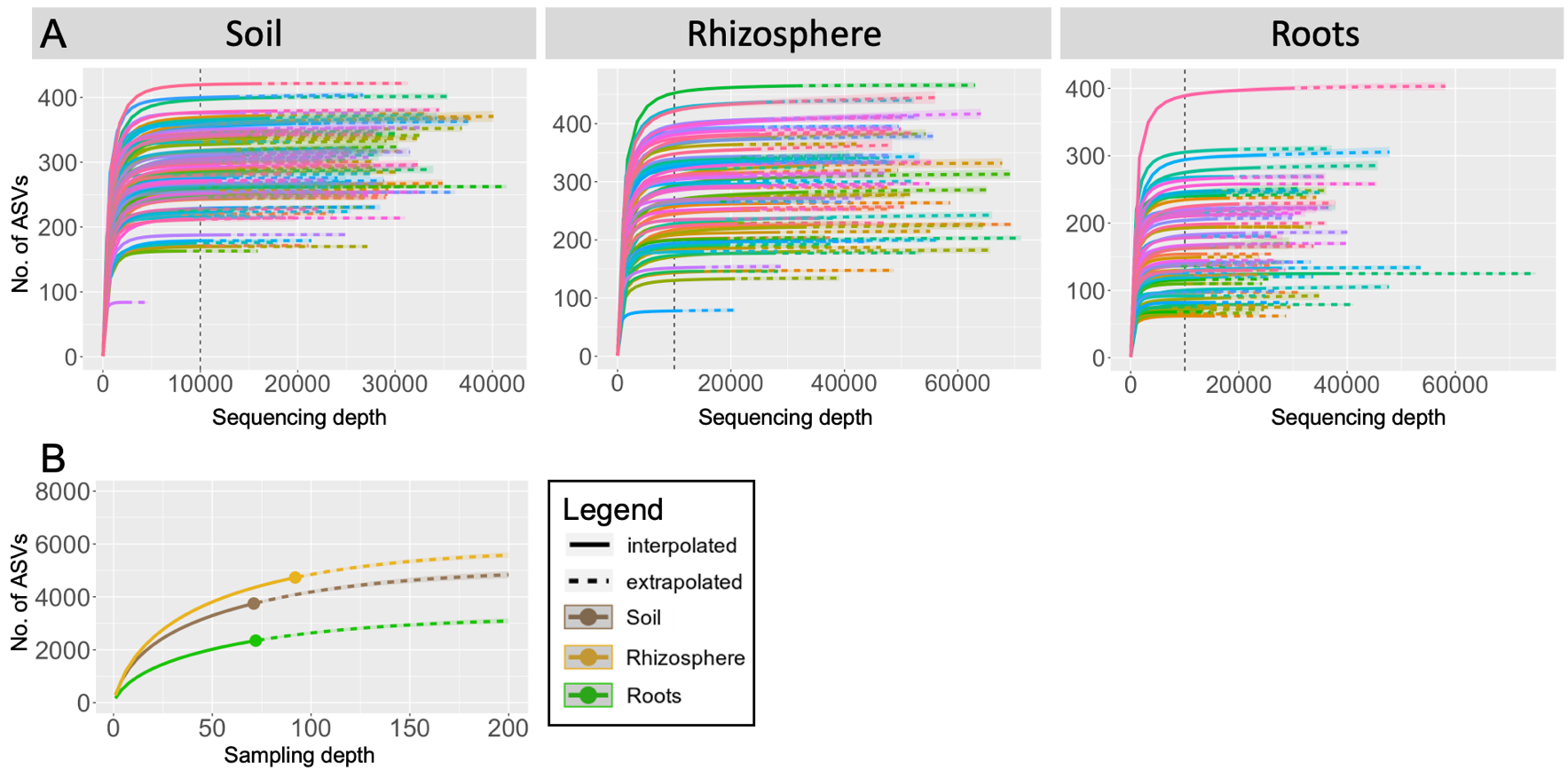
Figure S5. (A) Sequencing-depth-base rarefaction curves for the bacterial dataset and (B) sample-base rarefaction (solid line segments) and extrapolation (dotted line segments) curves for soil, rhizosphere and root samples. The vertical dotted lines in subfigures A represent the sampling depth of 10,000 sequences used for data normalization in soil, rhizosphere and root samples.


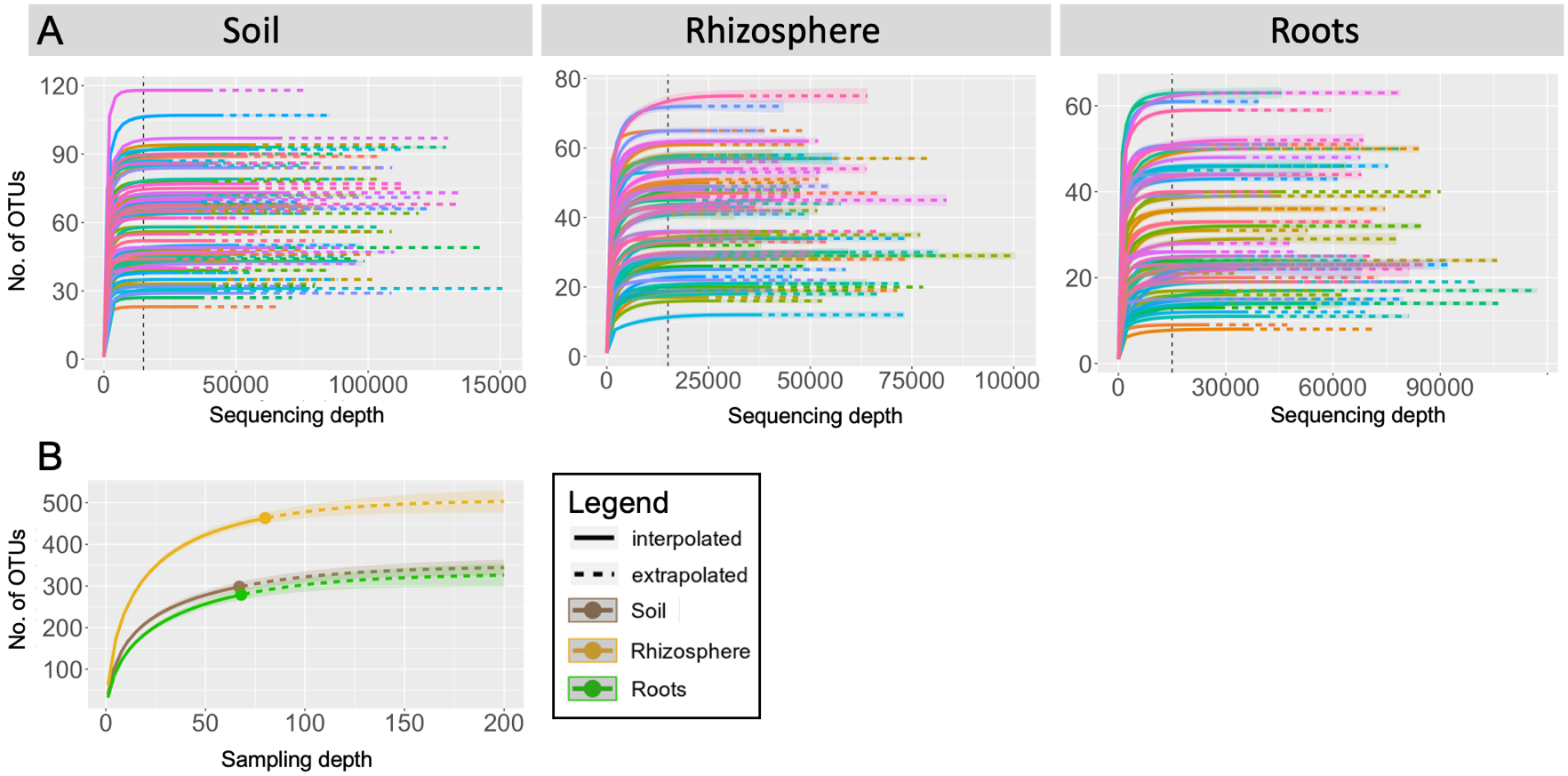
Figure S6. (A) Sequencing-depth-base rarefaction curves for the fungal dataset and (B) sample-base rarefaction (solid line segments) and extrapolation (dotted line segments) curves for soil, rhizosphere and root samples. The vertical dotted lines in subfigures A represent the sampling depth of the 15,000 sequences used for data normalization in soil, rhizosphere and root samples, respectively.


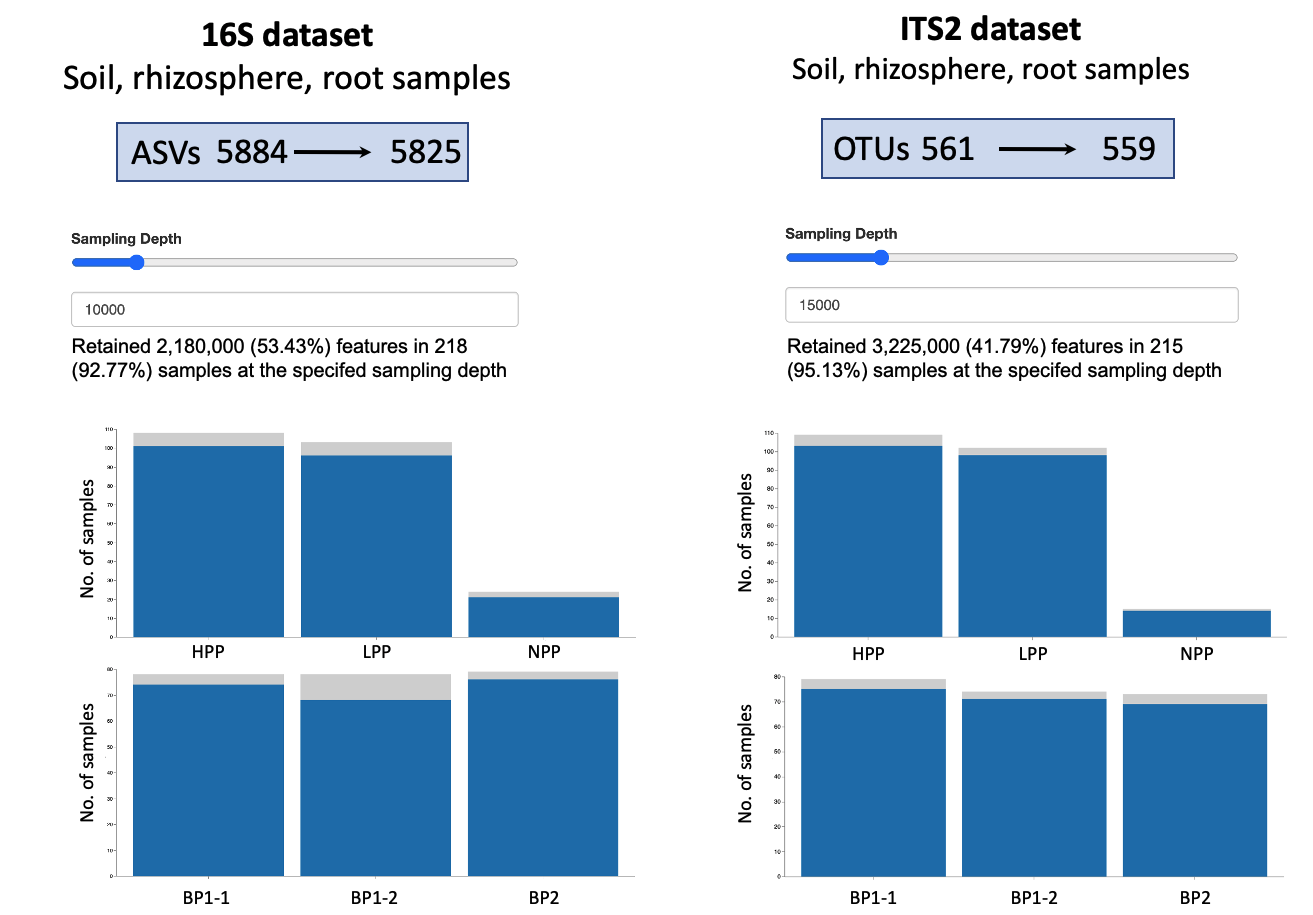
Figure S7. Number of samples removed after sequence rarefaction for each treatment and sampling site and its effect on the richness of bacterial ASVs and fungal OTUs.


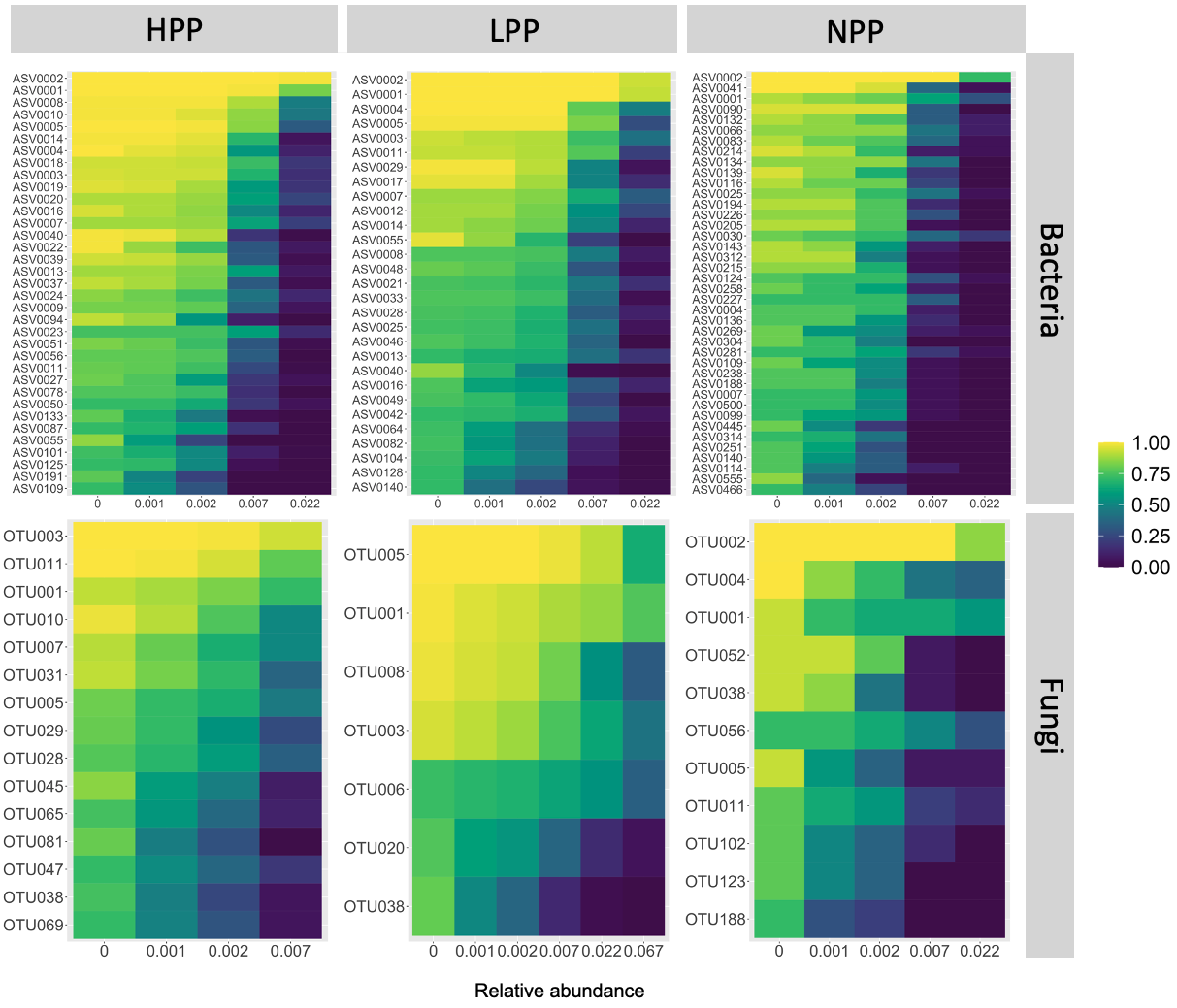
Figure S8. Core communities of bacteria (ASVs) and fungi (OTUs) recovered in samples from HPP (36 ASVs and 16 OTUs), LPP (29 ASVs and 7 OTUs), and NPP (43 ASVs and 9 OTUs). ASVs and OTUs are ranked from the most (top) to the least (bottom) prevalent in each compartment on the ordinate axis, and ordered by increasing relative abundance on the abscissa axis. Detection and prevalence thresholds were set to 0 and 70, respectively. The taxonomic assignments of the core ASVs and OTUs are presented in Tables S7 and S8.


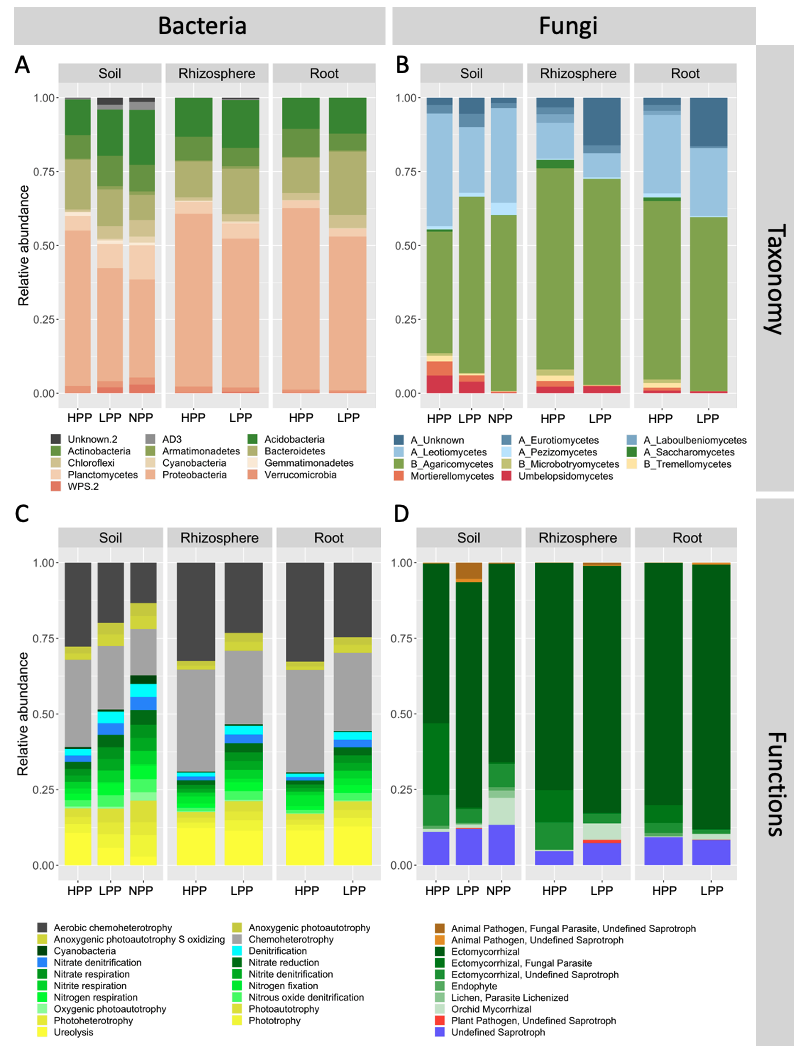
Figure S9. (A) and (B) Taxonomic profiles of the bacterial (phylum level) and fungal (class level) communities in soil, rhizosphere and root samples from high-productivity plots (HPP), low-productivity plots (LPP) and null-productivity plots (NPP). Phyla (bacteria) and classes (fungi) with a relative abundance of less than 1% were excluded. Fungal class names beginning with “A_” and “B_” are classes belonging to the phyla *Ascomycota* and *Basidiomycota*, respectively. (C) and (D) Bacterial and fungal ecological guilds. Functions with relative abundances below 4% (bacteria) and 1% (fungi) were excluded. These thresholds were chosen to obtain a manageable number of labels. For FUNGuild analysis, only guild assignments identified as “highly probable” and “probable” were considered. The effects of the productivity level treatments (high, low and null) on the structure of the functions were assessed using permutational multivariate analysis of variance (PERMANOVA) (Tables S1 and S2).
